# Detection and analysis of methicillin-resistant human-adapted sequence type 398 allows insight into community-associated methicillin-resistant *Staphylococcus aureus* evolution

**DOI:** 10.1186/s13073-018-0514-9

**Published:** 2018-01-29

**Authors:** Lei He, Hong-Xiang Zheng, Yanan Wang, Katherine Y. Le, Qian Liu, Jun Shang, Yingxin Dai, Hongwei Meng, Xing Wang, Tianming Li, Qianqian Gao, Juanxiu Qin, Huiying Lu, Michael Otto, Min Li

**Affiliations:** 1 Department of Laboratory Medicine, Renji Hospital, School of Medicine, Shanghai Jiaotong University, No. 160 Pujian Road, Shanghai, 200127 China; 2 Ministry of Education Key Laboratory of Contemporary Anthropology and Center for Evolutionary Biology, School of Life Sciences and Institutes of Biomedical Sciences, Fudan University, No. 2005 Songhu Road, Shanghai, 200438 China; 3 Pathogen Molecular Genetics Section, Laboratory of Bacteriology, National Institute of Allergy and Infectious Diseases, The National Institutes of Health, 50 South Drive, Bethesda, Maryland 20814 USA; 4 Shanghai Institute for Veterinary Drug & Feeds Control, No. 855 Hongjing Road, Shanghai, 201103 China; 5 Department of Laboratory Medicine, Shanghai Children’s Medical Center, Shanghai Jiaotong University School of Medicine, No. 1678 East Road, Shanghai, 200127 China

**Keywords:** *Staphylococcus aureus*, Community-associated MRSA, Livestock-associated MRSA, ST398, Virulence, Methicillin resistance

## Abstract

**Background:**

Severe infections with highly virulent community-associated methicillin-resistant *Staphylococcus aureus* (CA-MRSA) are a global problem. However, the molecular events defining the evolution of CA-MRSA are still poorly understood. MRSA of sequence type (ST) 398 is known to frequently infect livestock, while ST398 isolates infecting humans are commonly methicillin-susceptible or represent MRSA originating from livestock-associated (LA)-MRSA.

**Methods:**

We used whole genome sequencing of newly detected CA-MRSA ST398 isolates, in comparison to geographically matched LA-MRSA and methicillin-sensitive ST398, to determine their evolutionary history. Furthermore, we used phenotypic analyses including animal infection models to gain insight into the evolution of virulence in these CA-MRSA isolates. Finally, we determined methicillin resistance and expression of the methicillin resistance-conferring gene *mecA* and its penicillin-binding protein product, PBP2a, in a large series of CA-MRSA strains of divergent STs.

**Results:**

We report several cases of severe and fatal infections due to ST398 CA-MRSA. The responsible isolates showed the typical genetic characteristics reported for human-adapted methicillin-sensitive ST398. Whole genome sequencing demonstrated that they evolved from human-adapted, methicillin-susceptible clones on several different occasions. Importantly, the isolates had not undergone consistent genetic alterations or changes in virulence as compared to their methicillin-susceptible predecessors. Finally, we observed dramatically and consistently lower methicillin resistance and expression of the resistance gene *mecA*, as compared to hospital-associated MRSA strains, in a diverse selection of CA-MRSA strains.

**Conclusions:**

Our study presents evidence for the development of highly virulent human-adapted ST398 CA-MRSA isolates from methicillin-susceptible predecessors. Notably, our investigation indicates that, in contrast to widespread notions, the development of CA-MRSA is not necessarily associated with the acquisition of specific virulence genes or other virulence-increasing changes. Rather, our findings emphasize the importance of the CA-MRSA-characteristic staphylococcal cassette chromosome *mec* types, which provide only low-level methicillin resistance, for that process. Our findings are of particular importance for the diagnosis of CA-MRSA, inasmuch as they indicate that the presence of specific virulence genes cannot generally be used for that purpose.

**Electronic supplementary material:**

The online version of this article (doi:10.1186/s13073-018-0514-9) contains supplementary material, which is available to authorized users.

## Background

For decades, hospital-associated methicillin-resistant *Staphylococcus aureus* (HA-MRSA) infections have presented a major problem for public health systems all over the world, causing significant morbidity, mortality, and costs. More recently, highly virulent strains of MRSA have emerged that can infect healthy people outside of hospital settings (community-associated MRSA, CA-MRSA) [1]. How these new strains evolved to combine extraordinary virulence with methicillin resistance has remained largely unknown [2]. In particular, it has remained unclear whether there is a general molecular feature that CA-MRSA needed to acquire during evolution to develop that pathogenic potential. Previous models, predominantly based on research of pulsed-field type USA300, which caused a severe CA-MRSA epidemic in the USA [1], have focused on the acquisition of specific virulence factors. Among those, the prophage-encoded Panton-Valentine leukocidin (PVL) has received the most attention. While PVL increases virulence when present, highly virulent and epidemiologically successful PVL-negative CA-MRSA lineages have now emerged, casting doubt on a general importance of PVL for the evolution of CA-MRSA clones [2]. Furthermore, it has been noted that CA-MRSA isolates commonly have high expression of core genome-encoded toxins, such as α-toxin and phenol-soluble modulins (PSMs) [3], but the contribution of these toxins to CA-MRSA virulence is also variable [4]. It is generally accepted that all CA-MRSA clones have characteristic methicillin resistance-encoding mobile genetic elements (MGEs), namely staphylococcal cassette chromosome *mec* (SCC*mec*) of types IV and V [5, 6]. However, their role in CA-MRSA pathogenic success has remained poorly defined.

ST398 is a frequent cause of livestock-associated (LA) MRSA infections in many countries [7]. Occasionally, LA-MRSA ST398 is directly or indirectly transferred from livestock to humans [8], where it normally causes moderate and only occasionally severe infection [9–11]. In contrast, human-adapted ST398 isolates often cause severe and fatal infections and are genetically different from LA clones [7, 12, 13]. Some of the distinct genetic features of those isolates are reportedly important for the adaptation to humans as hosts, which may explain the more pronounced severity of infection they can cause in humans [12, 14]. Of note, almost all reported human-adapted ST398 isolates are methicillin-susceptible. Only two cases of human infections with ST398 MRSA have been reported, in which genetic analysis indicated that they are human-adapted rather than LA-MRSA transferred from livestock [14]. However, these isolates have not undergone more detailed phenotypic analysis. Furthermore, according to a recent study, LA-MRSA of ST398 appears to be able to undergo some sort of genetic adaptation to humans [15], similar to what has been described in detail for the bovine clonal complex 97 [16], but evidence for the development of entirely human-adapted ST398 MRSA that developed from human-adapted methicillin-susceptible predecessors by uptake of methicillin resistance has not yet been reported.

Here, we report several cases of severe and sometimes fatal infections with ST398 CA-MRSA without connection to livestock. Genomic analysis revealed that these highly infectious ST398 CA-MRSA isolates evolved from human-adapted methicillin-susceptible clones and do not represent transferred or adapted LA-MRSA. Furthermore, detailed genomic and phenotypic analysis of those isolates generated important information on CA-MRSA evolution. Namely, our findings cast doubt on the notion that the acquisition of virulence factors or a change in the expression of pre-existing toxin genes is a necessary step in that process. Rather, our results support the idea that what generally matters for the emergence of CA-MRSA is a low level of methicillin resistance gene expression.

## Methods

### Bacterial strains, growth conditions, and clinical definitions

*S. aureus* strains were grown in tryptic soy broth (TSB) (Oxoid) with 0.25% glucose or on tryptic soy agar plates at 37 °C. We collected and analyzed a total of 2048 clinical isolates from adult patients at Shanghai Renji hospital between 2005 and 2014. In the years 2005–2010, 100 isolates were randomly selected each year. In 2011, 2012, and 2014, all *S. aureus* isolates were included (no isolates from 2013 were obtained). Furthermore, all 185 *S. aureus* isolates obtained at Shanghai Children's Medical Center in 2012 were included. Bacterial strains of animal origin were collected from the Shanghai Animal Disease Prevention and Control Center, which is responsible for monitoring the epidemiology of animal infection in eastern China (see Additional file 1: Figure S1 for geographic matching of human and animal isolate sources). CA-*S. aureus* was defined as an isolate that was obtained either from an outpatient or from an inpatient (including those from general and urgent care and emergency rooms) ≤ 24 h after hospital admission and for which the patient lacked risk factors (contact with the hospital environment in the 6 months preceding the culture, *S. aureus* infection history or residence in a long-term care facility in the 12 months preceding the culture, presence of a central vascular catheter at the time of infection, and antibiotic use within at least 1 month preceding isolate collection, as determined by the review of medical records). An infection was considered invasive when isolates were isolated from otherwise sterile body sites. In this study, none of the patients from whom ST398 MRSA strains were isolated reported contact with animal farms or rearing animals in the 3 months preceding the culture. Of the eight ST398 CA-MRSA isolates obtained between 2005 and 2014, seven were further investigated in the present study after initial characterization of all eight isolates. One isolate could not be recovered for the in-depth analysis performed in our study.

### Antimicrobial resistance profiles

Antibiograms were determined by disc diffusion on Mueller-Hinton agar according to Clinical and Laboratory Standards Institute (CLSI) guidelines. The minimum inhibitory concentration (MIC) of oxacillin was determined by the broth microdilution MIC method, and interpretation of MIC results was based on 2015 CLSI guidelines.

### Molecular typing

Molecular typing was performed using multilocus sequence typing (MLST) as previously described [17]. The sequences of the polymerase chain reaction (PCR) products were compared with the existing sequences available at the MLST website (http://www.mlst.net). Newly detected STs were deposited to the MLST database.

### Whole genome sequencing of ST398 isolates and genome comparison

*S. aureus* whole genome sequencing was performed on an Illumina HiSeq 2500 sequencer (Illumina, San Diego, CA, USA) with 125-bp paired-end reads. The data generated from the Illumina platform were analyzed after quality control was performed. This involved processing the raw sequences by clipping the adapter sequences, removing non-A, G, C, T bases of the 5′ end, trimming low-quality sequencing reads (base quality with less than Q20), removing reads with > 10% of “N” base calls, and filtering small fragments of less than 25 bp.

Original sequencing reads were exported to Fastq files, and then bwa v0.7.12 [18] was used to align reads to the 2,872,582-base S0385 chromosome as a reference [GenBank:AM990992] to generate binary sequence alignment/map (BAM) files of *S. aureus* genomes [18]. The duplicate reads were removed by MarkDuplicates, implemented in Picard v1.82 (http://broadinstitute.github.io/picard/), and the mitochondrial DNA (mtDNA) sequences were locally realigned using Genome Analysis Toolkit (GATK) v1.2.59 [19]. Pileup files were generated by SAMtools v1.0.18 [18]. Consensus sequences were then obtained based on the pileup files. Genome bases were marked as unknown if they did not meet a minimum coverage of 10 × or if the minor allele was present in less than 75% of the base calls for that position. As a result, the 76 genomes were sequenced at an average depth of 419.42 (±165.67) ×, and the minimum coverage was 188.74 ×. Fastq files of 88 ST398 samples [14] were downloaded from GenBank, and the variants were also called using the preceding strategy. Additional contigs or genomes of nine ST398 strains were downloaded from GenBank; here, a coverage requirement was not applied in the variant calling.

### Phylogenetic analysis and time estimation

We used ST36 [GenBank:BX571856], determined by Price et al. as the most closely related non-CC398 ST [14], as an outgroup and aligned it to S0385 with the Multiple Alignment using Fast Fourier Transform (MAFFT) program [18]. In the phylogenetic analysis, overall single nucleotide polymorphisms (SNPs) had a markedly uneven distribution across the genome, largely related to whether the SNP resided in the core (present in all sample isolates) or accessory regions of the genome. We excluded accessory regions of the genome (the ~ 252,300-bp genome regions that 80% of the ST398 samples were missing and the ~ 123,000-bp putative horizontally transferred region). The accessory genome primarily comprises MGEs such as phages, transposons, SCC*mec*, and genomic islands. The presence of these elements was analyzed separately by analytical PCR. Because MGEs have an inherent potential for horizontal transfer between isolates, which could confound phylogenetic interpretations, we distinguished between the “core” and “noncore” genome for subsequent analysis.

We finally obtained 66,048 segregating sites from the alignment of 174 ST398 genome sequences and one ST36 sequence. The phylogeny tree was inferred by MrBayes v3.2.1 [20] using a general time-reversible (GTR) + G model and alignment of segregating sites. One million generations were performed with four chains (one cold chain and three hot chains), and the first 7000 generations were regarded as burn-in. Alternatively, PhyML v3.0 [21] with a GTR + G model was used. New haplogroups were defined according to the topology and assigned to each sample. We employed the Bayesian Evolutionary Analysis by Sampling Trees (BEAST) v1.8.0 program [22] to estimate the coalescence time of the haplogroups by the dated tip method. The result achieved using Phylogenetic Analysis by Maximum Likelihood (PAML) 4.7a [23] for time estimation was consistent. The mutation rate estimated by BEAST was 1.88 × 10^–6^/site/year (95% confidence interval (CI) of 1.62 × 10^–6^ – 2.11 × 10^–6^). That estimated by PAML was 1.67 ± 0 · 13 × 10^–6^/site/year. The coalescence time for all 174 ST398 strains was 60 years ago.

### Lysis of erythrocytes by culture filtrates

Supernatants were collected from bacterial cultures grown for 15 h. Hemolytic activities were determined by incubating samples with human red blood cells (2% v/v in Dulbecco’s phosphate-buffered saline, DPBS) for 1 h at 37 °C. Hemolysis was determined by measuring the optical density at 540 nm using an enzyme-linked immunosorbent assay (ELISA) reader. The assay was performed in triplicate.

### Measurement of PSMs, α-toxin, and penicillin-binding protein (PBP2a)

PSMs were measured in bacterial culture filtrates from cultures grown to stationary growth phase (8 h) by reversed-phase high-pressure liquid chromatography/mass spectrometry as previously described [24]. α-toxin was measured in precipitated culture filtrates (8 h) by western blotting using anti-α-toxin (1:1000, Abcam, Cambridge, MA, USA). For PBP2a detection, cell lysates were collected from bacterial cultures grown to stationary growth phase (8 h) and PBP2a amounts were measured by western blotting using anti-PBP2a (1:1000, BBI Solutions, Cardiff, UK). Anti-mouse IgG/horseradish peroxidase (HRP)-linked second antibody (1:1000, Tiangen) was used. PBP2a signals were normalized versus signals obtained for sortase A; α-toxin samples were normalized by the optical density of the cultures.

### Mouse skin abscess and lung infection models

Outbred, immunocompetent hairless female mice were used for the abscess model. Female BALB/c mice were used for the lung infection model. All mice were between 4 and 6 weeks of age at the time of use. *S. aureus* strains were grown to mid-exponential phase. Three mice were infected with each strain.

For the abscess model, mice were anesthetized with isoflurane and inoculated with 50 μl PBS containing 10^7^ live *S. aureus* or PBS alone in the right flank by subcutaneous injection. All mice were euthanized 48 h after infection. Length (L) and width (W) values were used to calculate the area of lesions with the formula L × W. Mouse skin tissue of the same size was taken from the abscess and PBS control groups and homogenated with glass beads with PBS-containing protease inhibitor cocktail (Roche) for cytokine detection. Quantikine ELISA mouse cytokine detection kits (R&D Systems, Minneapolis, MN, USA) were used to concentrate mouse tumor necrosis factor (TNF)-α and interleukin (IL)-6 from the skin homogenate.

For the lung infection model, 4 × 10^9^ colony-forming units (CFU)/40 μl *S. aureus* was pipetted into the nares of the anesthetized mice. All mice were euthanized 48 h after inoculation. The lungs from each group of animals were excised, washed with PBS, and homogenized in PBS, and *S. aureus* CFU/g lung tissue was determined by plating 100 μl homogenized lung tissue on TSB agar. The other homogenized lung tissue was used for cytokine detection.

### Analytical PCR

Analytical PCR to test for the presence of virulence genes was performed with the primers described previously [3]. The following primers were used for the *sak*, *scn*, and *chp* genes, respectively: sak-F, TGAGGTAAGTGCATCAAGTT; sak-R, TGTAATTAAGTTGAATCCAGGG; scnFw, TACTTGCGGGAACTTTAGC; scnRv, TTCGTCAATTTCGTTAT; chipsFw, CAACAGTTTTAGCATTAAGTTTTT; chipsRv, TTTTTCCAGGACCATTA.

### Quantitative reverse-transcription (qRT)-PCR

Overnight cultures were diluted 1:100 into 50 ml TSB and incubated at 37 °C with shaking at 200 rpm until grown to mid-exponential phase (4 h). Complementary DNA was synthesized from total RNA using the QuantiTect Reverse Transcription Kit (Qiagen) according to the manufacturer’s instructions. Oligonucleotide primers were designed using Primer Express. The primers used were gyrB-F, CAAATGATCACAGCATTTGGTACAG; gyrBR, CGGCATCAGTCATAATGACGAT; mecA-F, GTTAGATTGGGATCATAGCGTCATT; mecA-R, GCCTAATCTCATATGTGTTCCTGTAT. The resulting complementary DNA and negative control samples were amplified using the QuantiTect SYBR Green PCR Kit (Qiagen). Reactions were performed in a MicroAmp Optical 96-well reaction plate using a 7500 Sequence Detector (Applied Biosystems). Relative messenger RNA (mRNA) levels were calculated using *gyrB* as a control. All qRT-PCR experiments were performed in duplicate.

### Statistics

Statistical analysis was performed using GraphPad Prism v6.0. For the comparison of two groups, unpaired, two-tailed *t* tests were used; for three or more, one-way or two-way analysis of variance (ANOVA) was used, as appropriate. All error bars depict the standard deviation. Lines depict the mean.

## Results

We analyzed 125 CA-MRSA isolates from Shanghai hospitals obtained between 2005 and 2014. Among those, ST398 represented the third most frequently isolated lineage (eight cases), next to the predominant CA-MRSA lineage in China, ST59 [25] (79 cases) and ST1 (11 cases) (Table 1). All ST398 CA-MRSA isolates were negative for the *lukSF* genes encoding PVL and the PVL-encoding prophage sa2int. Remarkably, most (six of eight) of the ST398 CA-MRSA infection cases were severe (i.e., invasive) and two were fatal. These numbers, although too small for a statistically meaningful comparison, are exceptionally high, for example, when comparing to data we previously assembled for ST59 CA-MRSA in the same region [26], and emphasize the high virulence potential of the ST398 isolates (Tables 2 and 3).

**Table 1:** Sequence types of CA-MRSA isolates from this study

Sequence type (ST)	Number of cases
ST59	79
ST1	11
ST398	8
ST7	5
ST188	3
ST5	3
ST680	3
ST1821	2
ST239	2
ST88	2
ST181	1
ST25	1
ST86	1
ST9	1
ST4297	1
ST3969	1
ST4284	1

**Table 2:** Brief clinical reports of ST398 CA-MRSA cases

Patient/isolate number	Report
CA-MRSA-1	A 35-year-old previously healthy woman presented with 4 days of fever, productive cough, and right-sided pleuritic chest pain. On presentation, she was found to be febrile (38.8 °C), dyspneic, and tachypneic (RR 31 breaths/minute). Laboratory examination revealed peripheral leukocytosis with neutrophil predominance, and a chest radiograph showed diffuse patchy infiltrates. A bronchoscopy was performed and the bronchoalveolar lavage culture grew MRSA, which was subsequently identified as ST398. The patient was diagnosed with community-acquired staphylococcal pneumonia, and was treated with vancomycin, fosfomycin, and rifampicin for 21 days, and recovered
CA-MRSA-2	A 48-year-old healthy man presented with 1 week of fever, chills, productive cough, and chest discomfort. On examination, the patient was found to be febrile (38.7 °C), hypotensive (95/61 mmHg), and tachypneic (RR 28 breaths/minute). Laboratory examination revealed peripheral leukocytosis (white blood cells, WBC 13 × 10^9^/L), 78% of which were neutrophils. A chest radiograph demonstrated right middle lobe and bilateral lower lobe infiltrates. A bronchoscopy was performed and the bronchoalveolar lavage culture grew MRSA, which was identified as ST398. The patient was treated with vancomycin for 30 days and experienced recurrence of his staphylococcal pneumonia
CA-MRSA-3	A 54-year-old man presented with 2 days of productive cough, shortness of breath, and hemoptysis 12 h before admission. On examination, he was found to be febrile (39.5 °C), hypotensive (68/34 mmHg), tachycardic (136 beats/minute), and tachypneic (RR 46 breaths/minute). The patient was intubated and admitted to the intensive care unit. Laboratory examination revealed peripheral leukocytosis with neutrophil predominance, and a chest radiograph showed a large right lung infiltrate. Blood culture grew MRSA, typed as ST398. One week after admission, systemic infection occurred, signs of renal failure appeared, and MRSA ST398 could be detected in the peritoneal dialysate. The patient was treated with vancomycin, fosfomycin, and rifampicin. However, 2 weeks later, the patient expired due to sepsis and multiorgan failure
CA-MRSA-4	A 65-year-old man presented with 4 days of productive cough, nausea, vomiting, and diarrhea. On examination, the patient was found to be febrile (39.1 °C), hypotensive (78/45 mmHg), tachycardic (123 beats/minute), and tachypneic (RR 37 breaths/minute). Laboratory examination revealed leukocytosis with a left shift, and a chest radiograph demonstrated empyema. Sputum and blood cultures grew MRSA ST398. The patient was treated with vancomycin and linezolid for 3 weeks and recovered
CA-MRSA-5	A 2-year-old healthy boy presented with a purulent draining skin and soft tissue lesion on his back. On examination he had an elevated body temperature (37.9 °C). Culture was obtained from the drainage and grew MRSA, which was later identified as ST398. He was treated with clindamycin and linezolid for 1 week and recovered
CA-MRSA-6	A 3-year-old girl with rubella presented with a skin abscess on her left arm, and vomiting. On presentation, she was found to be febrile (39.7 °C). Laboratory examination revealed peripheral leukocytosis (WBC 13 × 10^9^/L, 84.6% of which were neutrophils), anemia (Hb 11.9 g/dL), and thrombocytopenia (platelets 55 × 10^9^/L). MRSA ST398 was isolated from the skin abscess as well as the blood stream. The patient was continuously treated with vancomycin, but subsequently developed infective endocarditis and a brain abscess and died 17 days after admission
CA-MRSA-7	A 21-year-old healthy woman presented with multiple purulent skin and soft tissue lesions on her back that developed over the course of 2 days. On examination, she was found to have a temperature of 38.0 °C. Laboratory examination showed peripheral leukocytosis (WBC 9.2 × 10^9^/L). MRSA later identified as ST398 was isolated from skin lesions. She underwent drainage of the soft tissue lesions and was treated with trimethoprim/sulfamethoxazole and clindamycin for 2 weeks with resolution of the skin abscesses
CA-MRSA-8 (not further investigated)	A 57-year-old healthy man presented with fever (40 °C), chills, chest pain, and petechial rash of 1 month in duration. On examination, he was found to have a 5+/6 apical systolic murmur that radiated to the axilla. A chest radiograph revealed bilateral pleural effusions. A transesophageal ultrasound showed mitral valve vegetations and mitral valve prolapse. MRSA identified as ST398 was isolated from the blood stream as well as the pleural fluid. The patient was treated with vancomycin, fosfomycin, and rifampicin and underwent mitral valve replacement surgery. He was discharged 2 weeks after his valve replacement surgery. The patient was readmitted to another hospital 4 months after discharge with an intracerebral abscess. MRSA was detected in the cerebrospinal fluid, which was not genotyped. The patient received vancomycin, fosfomycin, and rifampicin for 1 month, and was discharged.

**Table 3:** Characteristics of CA-MRSA ST398 isolates

Isolate	Year of isolation	Infection type	Invasiveness (isolation from otherwise sterile site)	Infection outcome	*spa* type	SCC*mec*	Penicillin^a^	Cefoxitin	Cefazolin	Levofloxacin	Erythromycin
CA-MRSA-1	2014	Respiratory	Invasive (isolated from bronchoalveolar lavage fluid)	Cured	t034	V	√	√	√		√
CA-MRSA-2	2014	Respiratory	Invasive (isolated from bronchoalveolar lavage fluid)	Recurrence	t034	V	√	√			√
CA-MRSA-3	2012	Respiratory, bacteremia	Invasive (isolated from blood and peritoneal dialysate)	Death	t034	V	√	√	√		√
CA-MRSA-4	2011	Respiratory, bacteremia	Invasive (isolated from sputum and blood)	Cured	t011	V	√	√	√		√
CA-MRSA-5	2012	SSTI	Non-invasive	Cured	t034	IV	√	√	√		
CA-MRSA-6	2012	SSTI, bacteremia	Invasive (isolated from blood)	Death	t571	V	√	√	√		√
CA-MRSA-7	2011	SSTI	Non-invasive	Cured	t034	None	√	√	√		√
CA-MRSA-8^b^	2012	Endocarditis, bacteremia	Invasive (isolated from pleural effusion and blood)	Recurrence	t034	ND	√	√	√		√

*SSTI* skin and soft tissue infection, *ND* not determined

^a^Resistance; no isolate was resistant to gentamicin, clindamycin, trimethoprim/sulfamethoxazole, fosfomycin, rifampicin, teicoplanin, vancomycin, or linezolid

^b^This isolate could not be recovered after the initial characterization shown in the table and was not included in further experiments in the study

**Fig. 1:**
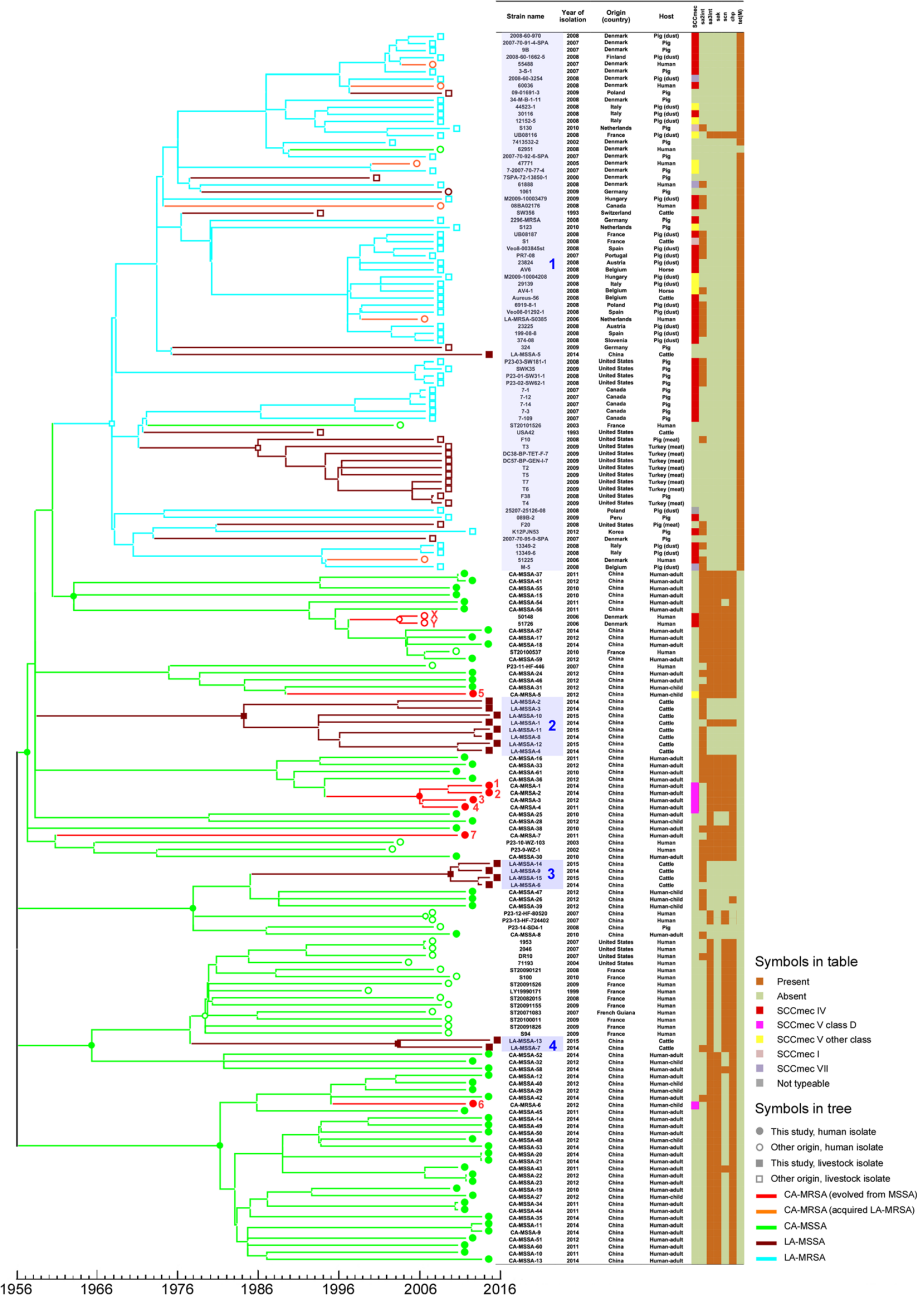
Phylogeny of ST398 isolates. We aligned 174 ST398 genome sequences (77 from our study, 88 from that by Price et al. [14], and 9 additional published sequences [12, 27–30]) and one ST36 sequence (outgroup). The phylogeny was inferred by MrBayes v3.2.1 [20], and time estimation was performed with the BEAST v1.8.0 program [22]. Isolate characteristics are shown on the *right*, including presence of SCC*mec* type and ST398 origin-defining genes. *1–7*, seven ST398 CA-MRSA isolates from this study; *X, Y,* two ST398 isolates of Chinese origin from the Price et al. study [14] that our analysis determined as CA-MRSA of human ancestry rather than transferred LA-MRSA strains. We also analyzed the presence of SNPs associated with the human and animal clades (branches *1–4*, marked in *blue*) and listed the corresponding SNPs in comparison to the human clade in Additional file 1: Table S1

To analyze the evolutionary position of the ST398 CA-MRSA isolates, we determined the genome sequences of seven of the eight ST398 CA-MRSA isolates, 53 ST398 methicillin-sensitive *Staphylococcus aureus* (MSSA) isolates obtained during the same time frame from the same hospitals, and 15 recent geographically matched livestock ST398 isolates. We also included in our genomic comparison all ST398 isolates whose genomes have been previously reported [12, 14, 27–30]. The phylogenetic tree we computed by the MrBayes method split in two overall clades, a human-adapted and a livestock-adapted clade, strongly suggesting, as noted by Price et al. [14], that the ST398 animal-adapted sublineage originated in humans as MSSA and then spread to livestock, where it subsequently acquired the methicillin resistance-harboring SCC*mec* cassette (Fig. 1). Computation by PhyML gave very similar results (Additional file 1: Figure S2). However, some animal isolates (branches 2, 3, and 4) clustered with the human clades, and no SNPs could be detected that generally distinguish human from animal ST398 isolates. Only the isolates in the most abundant animal branch 1, which corresponds to the isolates described by Stegger et al., showed 12 of the 13 SNPs described to be characteristic for animal ST398 isolates reported by those authors [31] (Additional file 1: Table S1). Notably, our phylogenetic analysis showed a much closer relationship of the Chinese CA-MRSA ST398 isolates with human-adapted MSSA ST398 (clade I) rather than LA-MRSA ST398 (clade II), indicating that they evolved from human-adapted MSSA predecessors by uptake of an SCC*mec* cassette. This notion is further confirmed by an analysis of the genetic determinants previously associated with human origin and adaptation of ST398 strains [12, 14], namely absence of the *tetM* resistance determinant and presence of a variant of prophage 3, containing the immune evasion complex (IEC) genes encoding the chemotaxis inhibitory protein (*chp*), staphylococcal complement inhibitor (*scn*), and staphylokinase (*sak*) (Fig. 1). These results are in accordance with the facts that we did not detect ST398 LA-MRSA among livestock isolates in Shanghai and that no patient with ST398 CA-MRSA infection reported animal contact.

Four of the ST398 CA-MRSA isolates (1–4) were closely related to each other, and age estimation based on SNP analysis indicated that they evolved from MSSA in about the year 2006. Isolates 5, 6, and 7 were unrelated to each other and evolved in about 1989, 1995, and 1961, respectively. The “old” isolate 7 was distinguished from the other six ST398 CA-MRSA clones in that its methicillin resistance was not linked to the presence of an SCC*mec* element, which is in accordance with recent findings showing that occasionally not only SCC*mec*-encoded *mecA* can confer β-lactam resistance [32]. The genome sequences of two previously published ST398 MRSA isolates [14] indicated that they also represent human CA-MRSA rather than transmitted LA-MRSA (Fig. 1). It is noteworthy that these two isolates also have a connection to China, as they are from Danish adoptees originating from China. Together, these results showed that human ST398 CA-MRSA evolved on multiple, unrelated occasions from human MSSA predecessors.

**Fig. 2:**
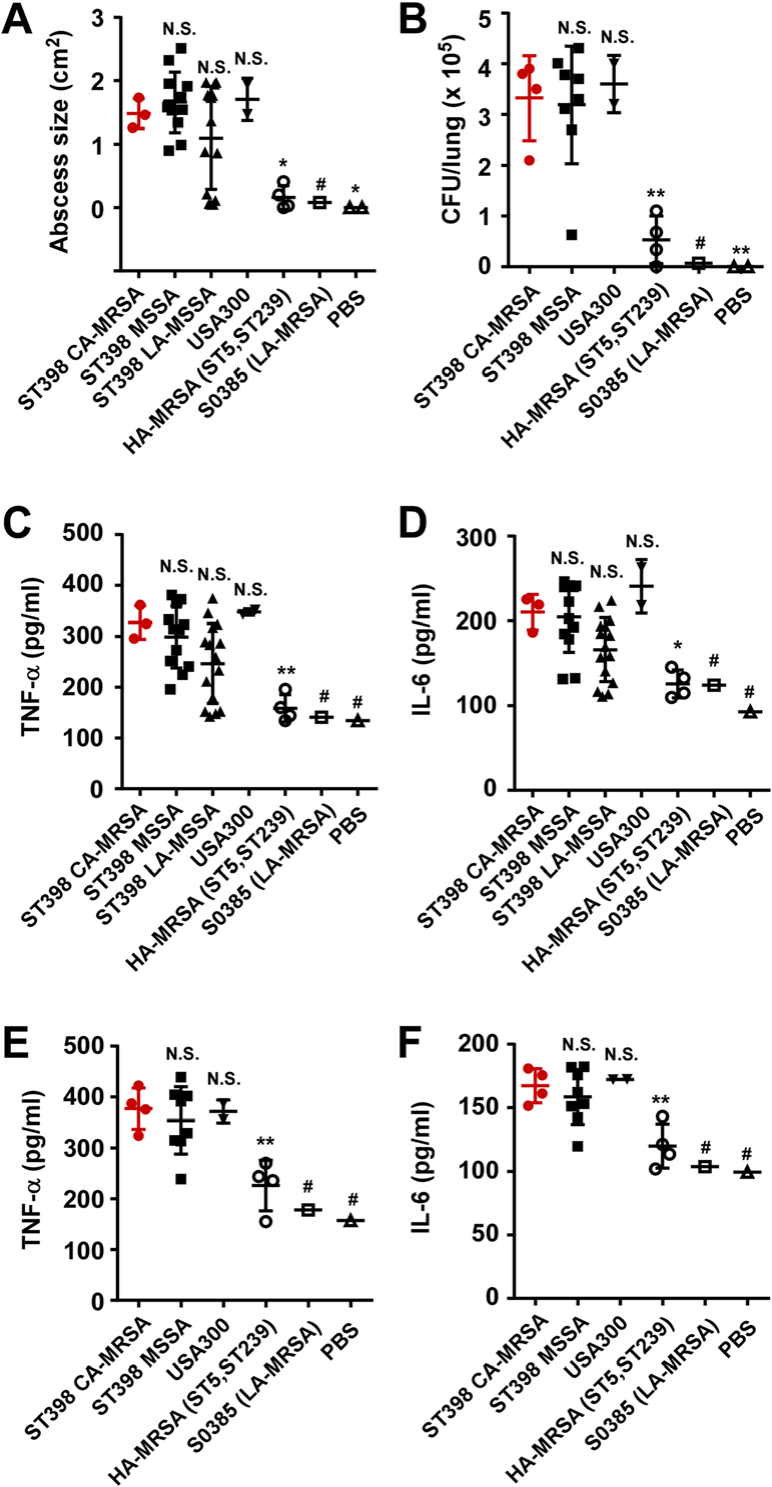
ST398 CA-MRSA virulence in a mouse model. The seven ST398 CA-MRSA isolates were compared with closely related ST398 MSSA, all ST398 LA-MSSA obtained for this study, and representative Chinese ST5 and ST239 HA-MRSA strains by in vivo analysis of virulence. In addition, two USA300 CA-MRSA strains (SF8300, LAC) and the genome-sequenced LA-MRSA S0385 standard strain were used in the comparisons. Isolate selection for the MSSA and HA-MRSA groups was performed, as in a previous study [26], by selecting isolates whose average PSM production was similar to that of the entire group and thus reflects the average virulence potential. Notably, for the in vivo infection studies, the ST398 CA-MRSA, MSSA, and ST5/ST239 HA-MRSA isolates were obtained from the corresponding type of human infection (respiratory and skin infection, respectively). See Additional file 1: Table S2 for isolates that were compared and their characteristics. **a** Mouse skin infection model. Mice received 10^7^ live *S. aureus* or PBS alone in the right flank by subcutaneous injection. Skin abscesses (length × width) were measured 48 h after infection. **b** Mouse lung infection model. We pipetted 4 × 10^9^ CFU/40 μl *S. aureus* into the nares of the anesthetized mice. All mice were euthanized 48 h after inoculation and CFU in the lung tissue were determined. **a**, **b** Three mice were infected with each strain; the shown symbols represent the average value obtained from three mice. **c**–**f** Inflammatory cytokine expression in ST398 CA-MRSA isolates in comparison. The inflammatory cytokines TNF-α and IL-6 were determined in skin (**c**, **d**) or lung tissue (**e**, **f**). **a**–**f** Statistical analysis is by one-way ANOVA with Dunnett’s post test versus the ST398 CA-MRSA group. *p < 0.05, **p < 0.01,*N.S.* not significant (*p* ≥ 0.05), # not included in the ANOVA as there is only one value

**Fig. 3:**
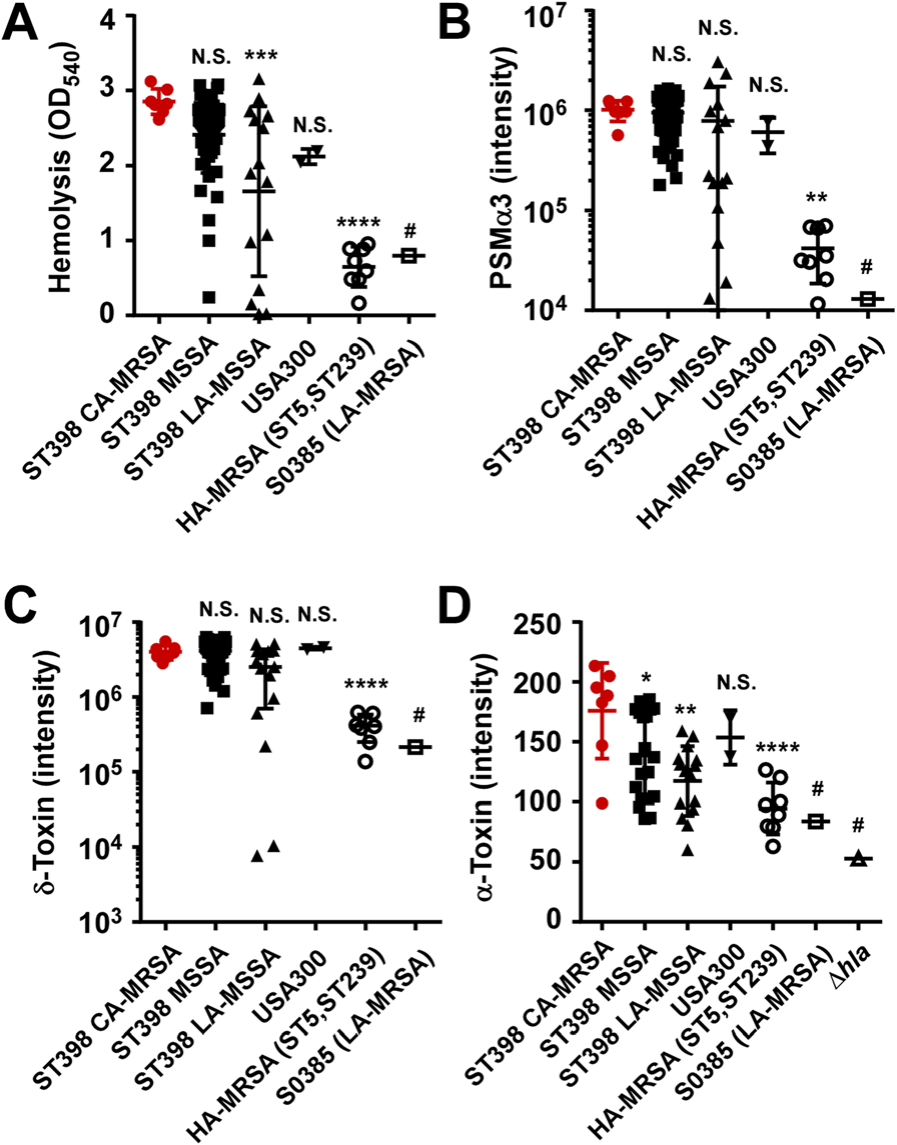
ST398 CA-MRSA virulence determinant expression. **a** Hemolysis (erythrocyte lysis). Hemolytic activities were determined by incubating culture filtrates with human red blood cells. **b**, **c** PSM production in culture filtrates. PSMα3 and δ-toxin are shown as examples. Measurement was by reversed-phase high-pressure liquid chromatography/mass spectrometry (RP-HPLC/MS). **d** α-toxin production in culture filtrates. Measurement was by western blot. Signals were measured by densitometry. *p < 0.05, **p < 0.01, ***p < 0.001, ****p < 0.0001, *N.S.* not significant (*p* ≥ 0.05), # not included in the ANOVA as there is only one value

The close relationship of the ST398 CA-MRSA and MSSA isolates made it possible to analyze the evolutionary changes leading to the development of virulent CA-MRSA clones in detail. Skin and lung infections are the most frequent infection types caused by *S. aureus*. Thus, we first compared virulence in mouse models of skin and lung infections of the ST398 CA-MRSA strains with closely related MSSA strains, geographically matched LA ST398 and representative isolates of predominant Chinese HA-MRSA strains (ST5, ST239) [33, 34], a standard, genome-sequenced LA-MRSA strain (S0385) [35], and the CA-MRSA clone USA300, which is especially notorious as a widespread source of CA-MRSA infections in the USA [36] (Additional file 1: Table S2). In both lung and skin infection models, the ST398 CA-MRSA isolates produced significantly more pronounced disease than the HA-MRSA and LA clones, at levels comparable to those elicited by USA300 and the human ST398 MSSA clones (Fig. 2a, b), with corresponding differences observed in the serum levels of inflammatory cytokines (Fig. 2c–f). We also measured cytolytic potential by analyzing lysis of human erythrocytes and determined expression of PSMs and α-toxin as important core genome-encoded virulence determinants (Fig. 3). All these analyses showed that the ST398 CA-MRSA isolates have the same high virulence as the closely related MSSA isolates, which is as pronounced as that found in the highly virulent USA300 CA-MRSA clone and strongly exceeds that of the predominant Chinese HA-MRSA lineages and the LA-MRSA clone S0385.

**Fig. 4:**
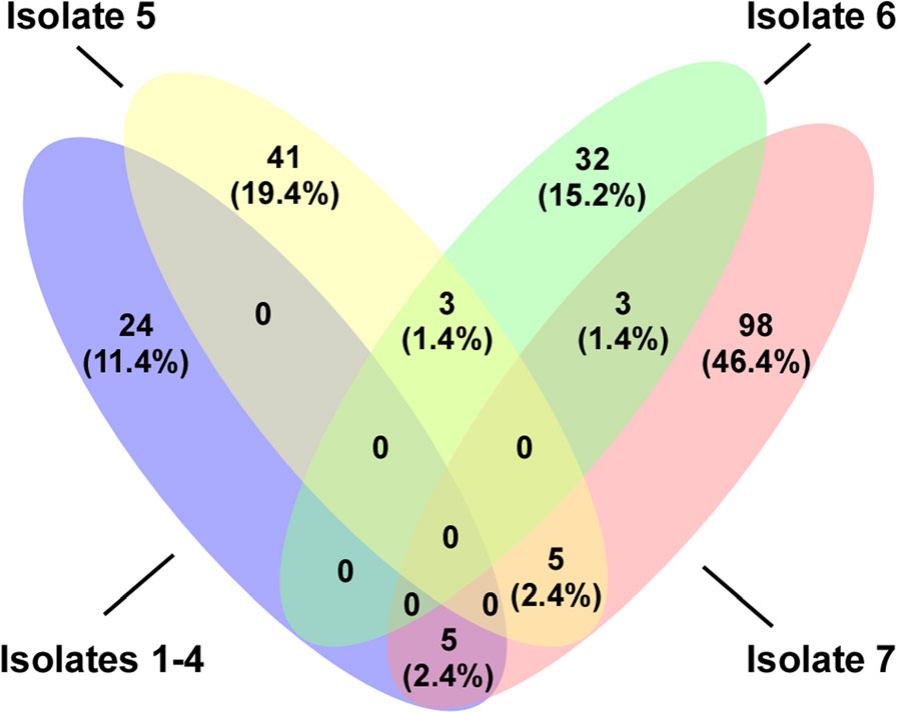
Lack of common genetic changes associated with ST398 CA-MRSA development. The figure shows a Venn diagram depicting the number of genes with non-synonymous SNPs common to the seven analyzed ST398 CA-MRSA isolates. All non-synonymous SNPs are listed in Additional file 1: Table S5. Note that there are no non-synonymous SNPs common to all isolates. Similarly, there were no consistent changes in virulence or antibiotic resistance genes or indels (Additional file 1: Tables S3, S4, and S6)

We then sought to determine whether the high virulence of the ST398 CA-MRSA clones was linked to the acquisition of specific virulence genes or due to other consistent genetic alterations. To that end, we performed a comprehensive analysis by analytical PCR testing for the presence of *S. aureus* virulence and antibiotic resistance genes, which frequently are located on MGEs (Additional file 1: Tables S3 and S4), and analyzed the core genome of the CA-MRSA isolates for non-synonymous SNPs and indels in protein-coding regions (Additional file 1: Tables S5 and S6). Remarkably, there was no consistent presence/acquisition of virulence or antibiotic (other than methicillin) resistance genes that would have discriminated the CA-MRSA from related MSSA clones, any common non-synonymous SNPs, or any consistent indel changes (Fig. 4, Additional file 1: Tables S3–S6). Thus, we detected no common genetic changes that are involved with the development of ST398 CA-MRSA from MSSA clones, in contrast to the notion that such processes are necessary for the evolution of CA-MRSA.

**Fig. 5:**
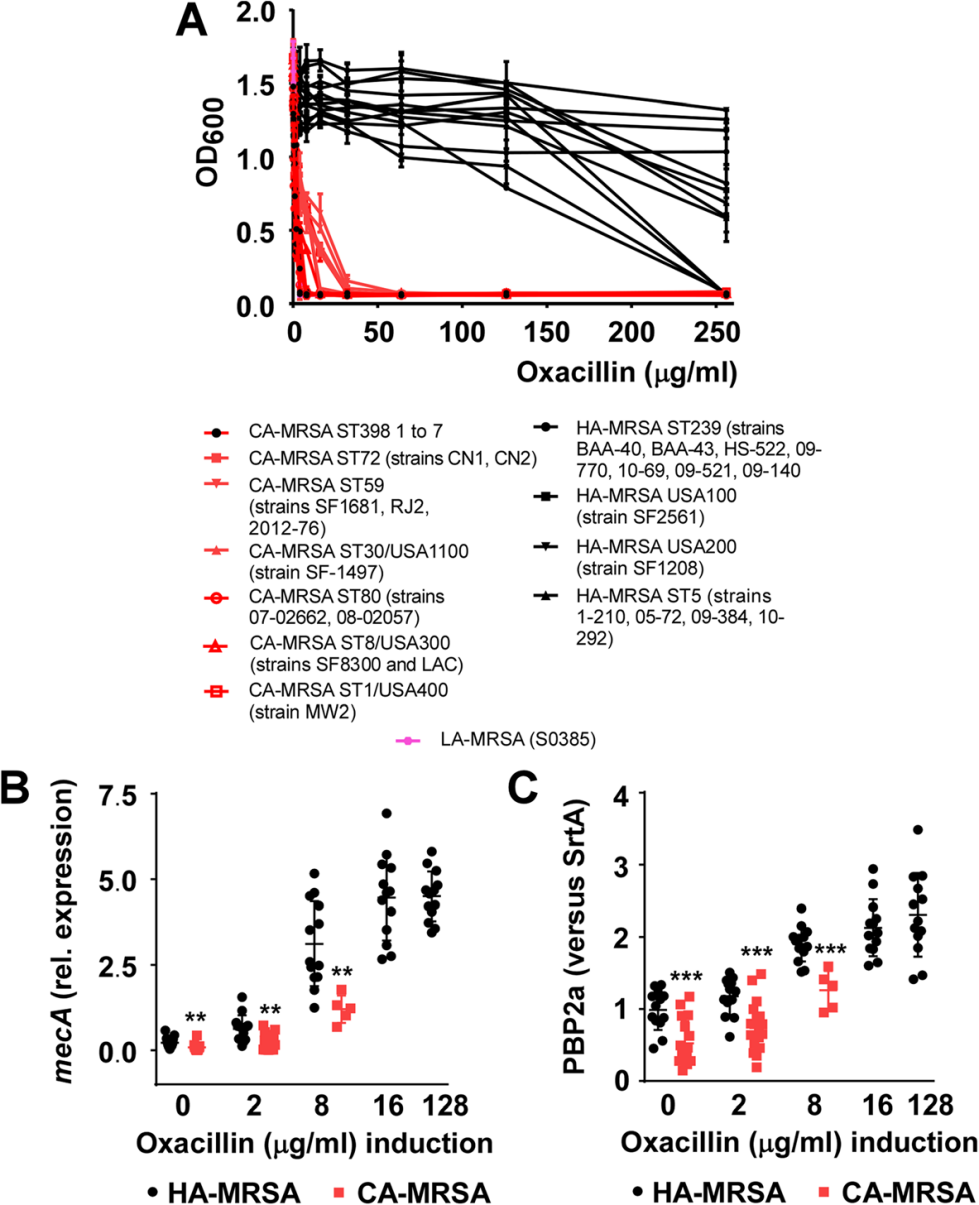
Methicillin resistance and *mecA* and PBP2a expression in CA- and HA-MRSA isolates. **a** Methicillin resistance (measured with oxacillin) by dilution method. **b** *mecA* expression levels by qRT-PCR in cultures grown to mid-exponential growth phase (4 h). **p < 0.01 (unpaired *t* test). **c** PBP2a levels by densitometry of western blots in stationary phase cultures (8 h) ***p < 0.001. Values are normalized versus sortase A signals obtained using the same samples. Strains used in **b**, **c** are the same as in **a**. Note that at induction levels of oxacillin higher than 4 μg/ml, only selected CA-MRSA strains grow and none grow at > 8 μg/ml

Considering previous findings that have shown repression of virulence by the methicillin resistance-encoding *mecA* gene [37, 38], these results prompted us to reassess a possible role of the CA-MRSA-characteristic SCC*mec* elements in the maintenance of CA-MRSA virulence. To that end, we investigated methicillin resistance in a large set of different CA- and HA-MRSA clones. We found striking and highly conserved differences (Fig. 5a). All HA-MRSA clones were strongly resistant, reaching minimum inhibitory concentration (MIC) values of 128 μg/ml and above, while methicillin resistance of all CA-MRSA clones barely reached an MIC of 4 μg/ml, the cutoff level used for MRSA classification. Furthermore, we measured expression of the *mecA* gene encoding the MRSA-characteristic penicillin-binding protein PBP2a on the transcript and protein level and found that, in agreement with our hypothesis, expression levels were significantly lower in all CA- as compared to HA-MRSA strains (Fig. 5b, c). These findings are in general agreement with those reported by Rudkin et al., who used a more limited set of isolates [38].

**Fig. 6:**
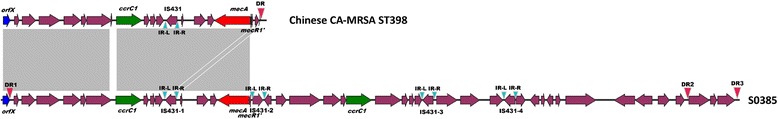
Novel CA-MRSA SCC*mec* element. *Top*, structure of the SCC*mec* element found in the Chinese ST398 CA-MRSA isolate with a class D (IS431-*mecA*-*mecR1*’) structure and one copy of *ccrC1*; *bottom*, class C (IS431-*mecA*-*mecR1*’-IS431) SCC*mec* element found in the LA-MRSA reference strain S0385, with two copies of *ccrC1*

**Fig. 7:**
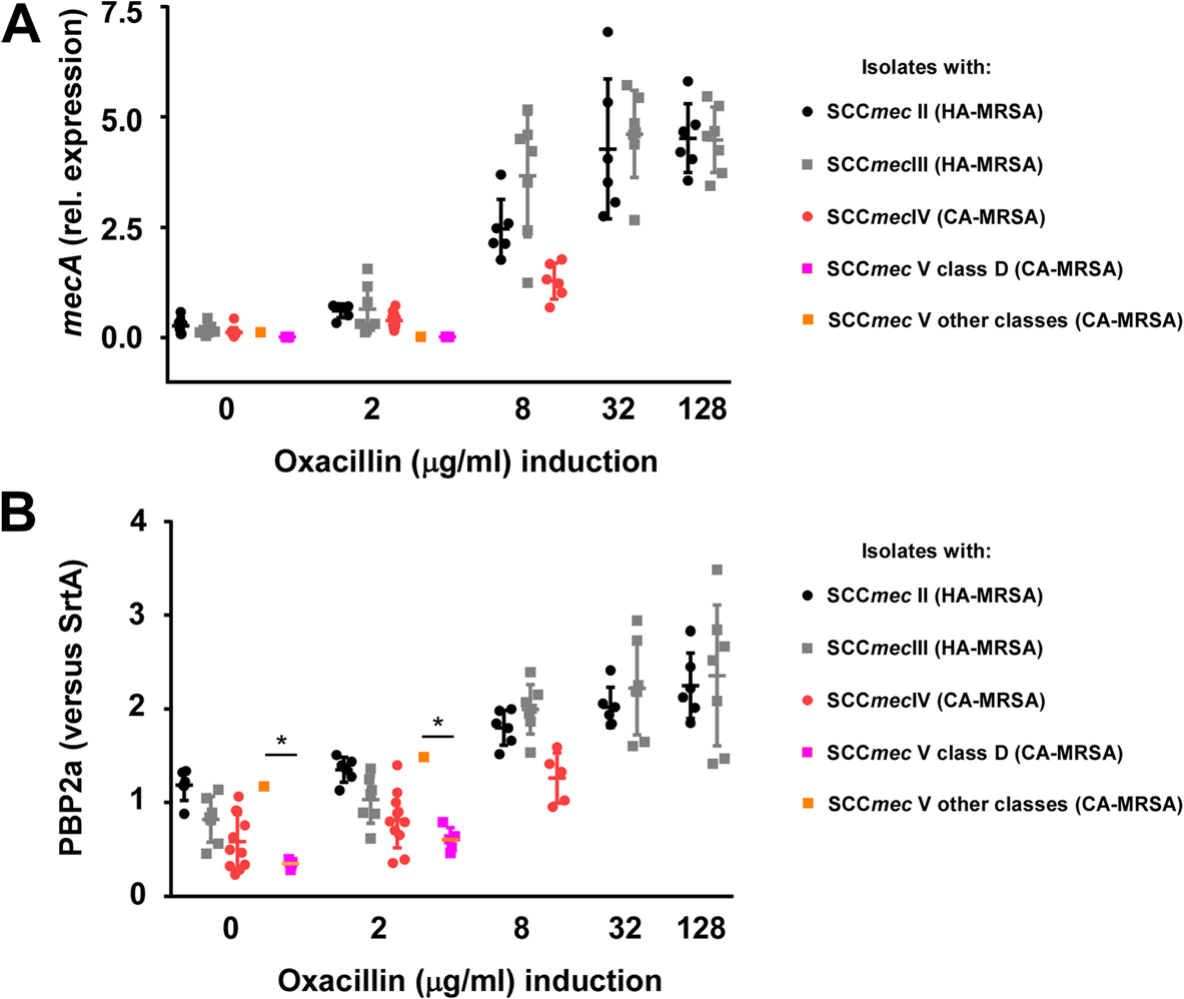
Expression of *mecA* and PBP2a in CA- and HA-MRSA strains by SCC*mec* type. Cultures were induced by different concentrations of oxacillin. **a** Expression of *mecA* was determined by qRT-PCR. **b** Expression of PBP2a was determined by western blot and values were normalized versus signals obtained for sortase A (*SrtA*). **a**, **b** *p < 0.05 (one-way ANOVA with Tukey post test). Note that at higher concentrations of oxacillin, only selected CA-MRSA strains grow

An interesting feature of the genomes of the Chinese ST398 CA-MRSA isolates was that we detected an SCC*mec* element not previously described in *S. aureus* in five of the seven sequenced isolates (isolates 1–4 and 6) (Fig. 6, Additional file 1: Table S7). It is a type V SCC*mec* element of class D, characterized by an IS431-*mecA*-*mecR1*’(truncated *mecR1*) composition, which has previously only been found in *S. caprae* [39]. This is in contrast to most other ST398 strains, including the reference strain S0385, which harbor class C SCC*mec* elements (composition IS431-*mecA*-*mecR1*’-IS431). Furthermore, it has only one copy of the *ccrC1* recombinase gene, while many ST398 isolates, including S0385, have two (Fig. 6). Isolate 5 from our study and the two ST398 MRSA isolates from the Chinese adoptees in Denmark have a type Vb (5C2&5) SCC*mec* element [GenBank:AB462393] [14], indicating independent acquisition of different SCC*mec* elements during the development of methicillin resistance in human-adapted ST398 MSSA predecessor strains and further substantiating that human-adapted ST398 CA-MRSA evolved on several independent occasions. Remarkably, in contrast to strains with other SCC*mec* types, strains with the CA-MRSA-characteristic SCC*mec* types only allowed growth at 2 μg/ml oxacillin, but not at higher concentrations. They also showed lower *mecA* and PBP2a expression at 0 and 2 μg/ml oxacillin. Isolates with the class D SCC*mec* type V showed particularly low *mecA* and PBP2a expression levels, even compared to other CA-MRSA-characteristic SCC*mec* types (Fig. 7). This novel CA-MRSA SCC*mec* type thus exemplifies particularly well that SCC*mec* elements in CA-MRSA confer only very low-level methicillin resistance and provides further support to the notion that the high virulence of CA-MRSA is inversely correlated with methicillin resistance levels.

## Discussion

The mechanisms associated with the evolution of highly virulent CA-MRSA isolates have been intensely debated. Most evidence has been derived from the analysis of the USA300 CA-MRSA isolates, which caused the most severe CA-MRSA epidemic up to this point [1]. USA300 CA-MRSA isolates characteristically contain the prophage harboring the genes encoding PVL [40], and many also have further MGEs that have been linked to the virulence potential of USA300 CA-MRSA isolates [41]. Other studies have noted that USA300 as well as several other CA-MRSA isolates show increased expression of core genome-encoded toxins such as PSMs and α-toxin [3]. Based on these findings, models explaining how the exceptional virulence of CA- as compared to HA-MRSA strains evolved always included the acquisition or increased expression of toxin and other virulence determinants [2]. In this study, we made use of our discovery of several severe community-associated infections due to virulent isolates of an ST not previously known as a source of CA-MRSA to analyze the evolutionary events associated with the development of CA-MRSA in detail, using a combined genomic and experimental approach. Our results with that ST indicate that virulence-increasing changes, in particular via the acquisition of virulence-harboring elements, are not necessary for the emergence of CA-MRSA. Rather, our analysis of methicillin resistance expression in a large number of different CA-MRSA isolates and STs supports the idea that CA-MRSA evolution is generally characterized by a very low methicillin resistance level. This is likely necessary to conserve the high virulence level of the MSSA predecessors of CA-MRSA, which is associated with the energy-consuming production of many toxins. Furthermore, our findings suggest that low PBP2a production (rather than, as previously hypothesized, their small size [42]) is the critical feature of the CA-MRSA-characteristic SCC*mec* elements. Our study has the limitation that we only found eight CA-MRSA isolates of the investigated ST in recent years. While this example shows that the acquisition of specific virulence-conferring genes is not absolutely necessary for the development of a CA-MRSA isolate, this does not exclude the possibility that in other CA-MRSA lineages such acquisition may significantly contribute to the virulence phenotype.

Our genomic and phenotypic investigation gives previously unavailable evidence to support the importance of the mechanistic model developed by Rudkin et al. [38] for CA-MRSA evolution. Namely, these authors showed that *mecA* represses the toxin regulator Agr, and thus low-level *mecA* expression as found in CA-MRSA is not accompanied by significant toxin repression. By showing that no genetic alterations accompany the acquisition of the low-level methicillin resistance SCC*mec* elements in the evolution of virulent CA-MRSA ST398, our study emphasizes the central importance of this mechanism in maintaining the high virulence of the MSSA predecessors during CA-MRSA evolution,

Furthermore, our findings are of great importance for the epidemiology of the ST398 lineage of *S. aureus*. While ST398 became infamous as a frequent source of livestock MRSA infections, this is the first detailed report on truly human ST398 CA-MRSA, i.e., CA-MRSA which developed from human-adapted predecessors. Clinical and virulence data indicate that this new CA-MRSA ST has the potential for serious and fatal infections and should be monitored for its potential spread. With the epidemiological success of CA-MRSA isolates not being completely understood, but likely not solely linked to their virulence potential, it is difficult to say at present whether ST398 CA-MRSA will cause an epidemic as seen with USA300 or ST59 in the USA and China, respectively.

Our results have important implications for attempts that have been made to diagnose CA-MRSA using genetic markers. PVL has frequently been suggested as such a marker, but the discovery of PVL-negative CA-MRSA has made such an approach obsolete. Our findings show that PVL is not necessarily replaced by other acquired virulence factors in other, PVL-negative CA-MRSA STs, emphasizing the notion that virulence genes can generally not be used as genetic markers to distinguish CA- from HA-MRSA. On the other hand, analysis of the CA-MRSA-characteristic SCC*mec* elements does not allow verification of the high virulence potential of a typical CA-MRSA isolate. Our findings thus emphasize that the definition of a CA-MRSA isolate should always be clinical, and that analysis of the potential threat of specific CA-MRSA isolates in the hospital setting is not easily amenable to genetic testing. Furthermore, the commonly low methicillin resistance may potentially be of value in considering alternative ways to treat CA-MRSA infections. Finally, the relative ease of CA-MRSA development illustrated herein by the multiple recent SCC*mec* uptake events leading to new CA-MRSA clones suggests that the development of novel, highly virulent CA-MRSA lineages is a likely scenario.

## Conclusions

In this article we report the detection of CA-MRSA isolates of ST398 and demonstrate that they evolved by the uptake of SCC*mec* elements from human MSSA without the accompanying uptake of any additional genetic factors, including most notably virulence factors. These findings support the notion that the evolution of CA-MRSA is not necessarily dependent on the uptake of specific genetic factors other than the CA-MRSA-characteristic SCC*mec* elements, which characteristically express the methicillin resistance gene *mecA* at a low level. This is likely to balance the energetic requirements associated with the high expression of virulence factors that is present in the predecessor strains and does not significantly change in the emerged CA-MRSA isolates.

Our findings emphasize that the definition of CA-MRSA can only be clinical, and no specific virulence genes can be used to unambiguously distinguish CA-MRSA isolates. Whether the highly virulent ST398 CA-MRSA isolates that we detected will spread is likely dependent on not yet fully understood factors, such as those contributing to colonization, and will thus require further monitoring. Further studies will also be required to investigate whether the generally low methicillin resistance level of CA-MRSA can be of clinical use.

## Additional file

Additional file 1: Figure S1. Geography of sources of animal and human isolates. **Figure S2**. Phylogeny of ST398 isolates according to PhyML method. **Table S1**. Non-synonymous substitutions that define the four animal ST398 branches. **Table S2**. Characteristics of isolates used for virulence experiments. **Table S3**. Presence of virulence genes in analyzed isolates. **Table S4**. Presence of antibiotic resistance in analyzed isolates. **Table S5**. Non-synonymous SNPs (as compared to the MRSA ancestor node and the closest MSSA neighbors) in ST398 CA-MRSA isolates. **Table S6**. Indel genetic changes in protein-coding regions of ST398 CA-MRSA isolates. **Table S7**. Open reading frames (ORFs) in the novel ST398 *SCCmec* type. (PDF 2409 kb)

## Abbreviations

CA: Community-associated
CC: Clonal complex
HA: Hospital-associated
LA: Livestock-associated
MGE: Mobile genetic element
MIC: Minimum inhibitory concentration
MLST: Multilocus sequence typing
MRSA: Methicillin-resistant *Staphylococcus aureus*
MSSA: Methicillin-sensitive *Staphylococcus aureus*
PBP: Penicillin-binding protein
PSM: Phenol-soluble modulin
PVL: Panton-Valentine leukocidin
SCC: Staphylococcal cassette chromosome
SNP: Single nucleotide polymorphism
ST: Sequence type
