# Supplementary material for: Detection and analysis of methicillin-resistant human-adapted sequence type 398 allows insight into community-associated methicillin-resistant Staphylococcus aureus evolution
Source: Genome Med. 2018 Jan 29;10:5. doi: 10.1186/s13073-018-0514-9 (PMC5789642; doi:10.1186/s13073-018-0514-9)
Supplement: Additional file 1: Figure S1. — Geography of sources of animal and human isolates. Figure S2. Phylogeny of ST398 isolates according to PhyML method. Table S1. Non-synonymous substitutions that define the four animal ST398 branches. Table S2. Characteristics of isolates used for virulence experiments. Table S3. Presence of virulence genes in analyzed isolates. Table S4. Presence of antibiotic resistance in analyzed isolates. Table S5. Non-synonymous SNPs (as compared to the MRSA ancestor node and the closest MSSA neighbors) in ST398 CA-MRSA isolates. Table S6. Indel genetic changes in protein-coding regions of ST398 CA-MRSA isolates. Table S7. Open reading frames (ORFs) in the novel ST398 SCCmec type. (PDF 2409 kb) [file 13073_2018_514_MOESM1_ESM.pdf]

Detection and analysis of methicillin-resistant human-adapted sequence type 398 allows insight into community-associated methicillin-resistant *Staphylococcus aureus* evolution

Lei He, Hong-Xiang Zheng, Yanan Wang, Katherine Y. Le, Qian Liu, Jun Shang, Yingxin Dai, Hongwei Meng, Xing Wang, Tianming Li, Qianqian Gao, Juanxiu Qin, Huiying Lu, Michael Otto, Min Li

Additional Material

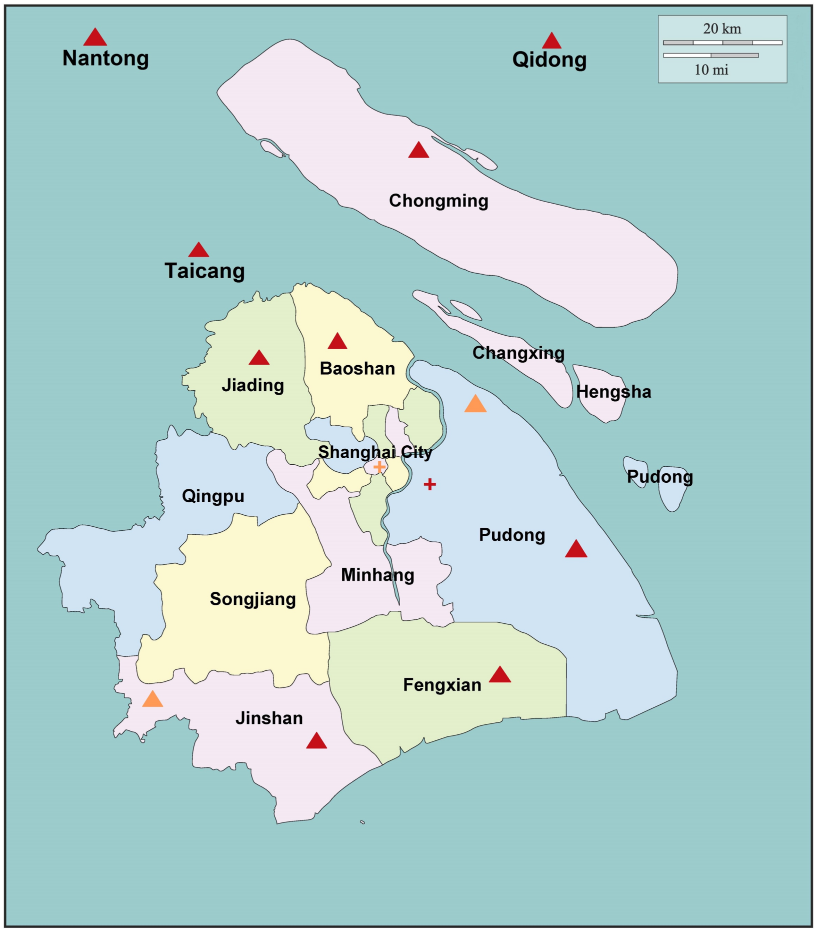

Figure S1: Geography of sources of animal and human isolates. Red and orange crosses mark the East and West sections, respectively, of Shanghai Renji hospital. Isolates 1, 2, 3, 6, and 8 were from the East and 4, 5, and 7 from the West section. Animal (cattle) isolates were from 15 dairy farms located around Shanghai (red triangles). Orange triangles mark locations with two dairy farms in the same district. The map background is from www.dmap.com.

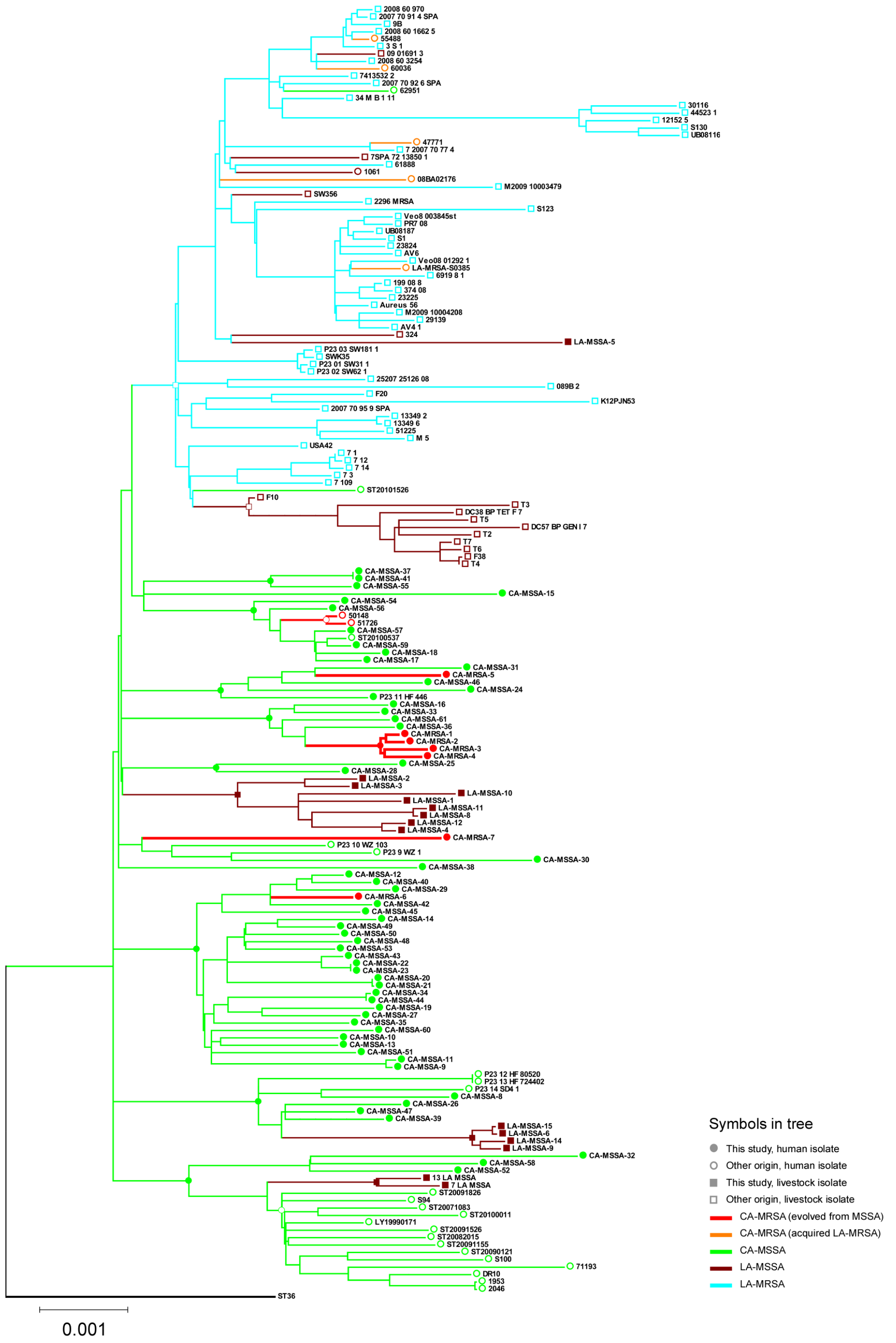

Figure S2. Phylogeny of ST398 isolates according to PhyML method
174 ST398 genome sequences (77 from our study, 88 from that by Price et al. [14], and nine additional published sequences [12, 27-30]) and one ST36 sequence (outgroup) were aligned. The phylogeny was inferred by PhyML.

Table S1. Non-synonymous substitutions that define the four animal ST398 branches

Branch[1]	Genomic position[2]	Gene	Amino acid change	
			Human clade	Livestock clade
Branch 1	C9319T[3]	*gyrA*	Ala	Val
Branch 1	G237199A	SAPIG0218	Val	Ile
Branch 1	G244322A[3]	SAPIG0223	Met	Ile
Branch 1	C422922T	SAPIG0406	Gln	*
Branch 1	C425594T[3]	SAPIG0409	Pro	Ser
Branch 1	C551946T[3]	SAPIG0537	Pro	Ser
Branch 1	G670004T	SAPIG0636	Gly	Val
Branch 1	G732619T[3]	SAPIG0698	Leu	Ile
Branch 1	G868021T	SAPIG0827	Arg	Leu
Branch 1	G1445215T	*trpF*	Val	Phe
Branch 1	G1518366T[3]	SAPIG1434	Ala	Glu
Branch 1	C1524032T[3]	SAPIG1434	Met	Ile
Branch 1	C1643719A	SAPIG1555	Gly	Cys
Branch 1	G1934659A[3]	SAPIG1823	Pro	Leu
Branch 1	A2168763G	*ilvB*	Glu	Gly
Branch 1	C2395959T[3]	SAPIG2317	Pro	Leu
Branch 1	G2458888A	*hutU*	Pro	Ser
Branch 1	A2512346G	SAPIG2434	His	Arg
Branch 1	G2597585A[3]	SAPIG2511	Gly	Glu
Branch 1	G2649984A	SAPIG2563	Val	Ile
Branch 1	A2703901C	SAPIG2613	Val	Gly
Branch 1	A2803205T	SAPIG2699	Met	Lys
Branch 1	G2805707A[3]	SAPIG2701	Arg	Cys
Branch 1	T2806556A[3]	SAPIG2701	Thr	Ser
Branch 2	C138935T	SAPIG0132	Ala	Val
Branch 2	C158846T	*deoB*	Pro	Leu
Branch 2	G178981T	SAPIG0170	Cys	Phe
Branch 2	C224169A	SAPIG0208	Gln	Lys
Branch 2	A263045G	SAPIG0238	Asp	Gly
Branch 2	C471579T	*ahpF*	Ala	Thr
Branch 2	G539078A	SAPIG0526	Gly	Asp
Branch 2	T551764A	SAPIG0537	Ile	Asn
Branch 2	C667145A	SAPIG0635	Thr	Lys
Branch 2	G771474A	SAPIG0737	Val	Ile
Branch 2	A1022550T	*clpB*	Lys	Met
Branch 2	A1075768T	*comK*	Leu	Met
Branch 2	T1214189A	SAPIG1161	Asn	Ile
Branch 2	A1361347G	*mutS*	Thr	Ala
Branch 2	G1375055T	SAPIG1309	Asp	Tyr
Branch 2	C1416112G	SAPIG1350	Thr	Arg
Branch 2	G1515870T	SAPIG1434	Ala	Glu

Branch 2	C1533358T	SAPIG1434	Gly	Ser
Branch 2	G1644816T	SAPIG1555	Ser	*
Branch 2	G1659686T	*zwf*	Arg	Leu
Branch 2	T1661323A	SAPIG1573	Glu	Val
Branch 2	C1661324T	SAPIG1573	Glu	Lys
Branch 2	C1683337T	SAPIG1595	Asp	Asn
Branch 2	G1754196A	SAPIG1673	Ala	Val
Branch 2	C1861256G	SAPIG1765	Arg	Thr
Branch 2	C1869455T	SAPIG1773	Val	Ile
Branch 2	C2032268T	*hemL*	Ala	Val
Branch 2	T2191579G	*acpS*	Gln	His
Branch 2	C2318899A	SAPIG2234	Met	Ile
Branch 2	G2382021A	SAPIG2307	His	Tyr
Branch 2	G2443671C	SAPIG2367	Ala	Gly
Branch 2	G2454873A	SAPIG2380	Arg	Cys
Branch 2	G2478525A	SAPIG2404	Pro	Ser
Branch 2	T2591020C	SAPIG2505	Asp	Gly
Branch 2	T2630547C	SAPIG2548	Val	Ala
Branch 2	T2740727G	SAPIG2652	Lys	Gln
Branch 2	G2741453T	SAPIG2652	Arg	Ser
Branch 2	T2806708C	SAPIG2701	Tyr	Cys
Branch 3	C251308A	SAPIG0229	Pro	Thr
Branch 3	G431170A	SAPIG0415	Met	Ile
Branch 3	G437147A	SAPIG0421	Pro	Ser
Branch 3	G439406C	SAPIG0424	Ala	Pro
Branch 3	C476859T	SAPIG0462	Asp	Asn
Branch 3	G515707T	SAPIG0502	Lys	Asn
Branch 3	G590185A	*mfd*	Asp	Asn
Branch 3	G603574A	*folP*	Gly	Asp
Branch 3	G657511A	SAPIG0625	Met	Ile
Branch 3	A709291G	SAPIG0673	Ile	Val
Branch 3	A709447G	SAPIG0673	Thr	Ala
Branch 3	A709600C	SAPIG0673	Thr	Pro
Branch 3	A709609G	SAPIG0673	Lys	Glu
Branch 3	T793228A	SAPIG0760	*	Tyr
Branch 3	T793229A	SAPIG0760	*	Leu
Branch 3	C793707T	SAPIG0760	Asp	Asn
Branch 3	G807084A	SAPIG0772	Ala	Thr
Branch 3	C934628A	SAPIG0894	Ser	Arg
Branch 3	G983621C	*argH*	Thr	Arg
Branch 3	C1028833A	*fabF*	Gln	Lys
Branch 3	A1125765T	SAPIG1073	Tyr	Asn
Branch 3	A1126083T	SAPIG1073	Phe	Ile
Branch 3	T1127066A	SAPIG1074	Lys	Asn
Branch 3	C1172567T	SAPIG1117	Gly	Asp
Branch 3	C1241941A	*ileS*	Arg	Ser

Branch 3	G1254760A	*carB*	Gly	Ser
Branch 3	C1288045T	*smc*	Thr	Met
Branch 3	T1307227C	*topA*	Tyr	His
Branch 3	T1312723G	*hslU*	Phe	Cys
Branch 3	G1320677T	*proS*	Leu	Phe
Branch 3	C1345088T	SAPIG1282	Ser	Phe
Branch 3	C1355625A	SAPIG1292	Pro	His
Branch 3	C1383901T	SAPIG1317	Ala	Thr
Branch 3	G1394559T	SAPIG1330	Asp	Tyr
Branch 3	T1488221A	*sucB*	Glu	Asp
Branch 3	C1489751T	*sucA*	Asp	Asn
Branch 3	C1496804A	SAPIG1420	Gly	Cys
Branch 3	T1522101A	SAPIG1434	Asn	Ile
Branch 3	C1525969T	SAPIG1434	Val	Ile
Branch 3	A1568691C	SAPIG1459	Ser	Ala
Branch 3	C1701589T	SAPIG1617	Arg	His
Branch 3	A1703938T	SAPIG1620	Phe	Tyr
Branch 3	T1726017C	*prmA*	Asn	Ser
Branch 3	T1742358C	*aroE*	Ile	Val
Branch 3	C1747881G	SAPIG1667	Gly	Ala
Branch 3	G1829446A	SAPIG1744	His	Tyr
Branch 3	C1830637T	SAPIG1744	Val	Ile
Branch 3	G1832632C	SAPIG1745	Pro	Ala
Branch 3	C1857809A	*ald*	Gly	Val
Branch 3	C1874811A	*nagE*	Glu	*
Branch 3	G1879214C	*tyrS*	Ile	Met
Branch 3	C1924178A	SAPIG1812	Lys	Asn
Branch 3	G1940450T	SAPIG1829	Lys	Asn
Branch 3	C1945951T	SAPIG1836	Trp	*
Branch 3	A1955816G	SAPIG1845	Cys	Arg
Branch 3	C1973750G	SAPIG1869	Pro	Ala
Branch 3	T2009509G	SAPIG1914	Thr	Pro
Branch 3	T2049348A	SAPIG1972	Leu	Phe
Branch 3	A2082695T	SAPIG2004	Ile	Phe
Branch 3	T2144151G	SAPIG2069	Lys	Gln
Branch 3	A2250704T	*coaA*	Glu	Asp
Branch 3	T2272531A	SAPIG2195	Arg	Ser
Branch 3	A2288233T	SAPIG2211	Asn	Tyr
Branch 3	C2439187A	SAPIG2362	Met	Ile
Branch 3	A2540813G	SAPIG2462	Gln	Arg
Branch 3	G2541049A	SAPIG2462	Asp	Asn
Branch 3	G2578126A	SAPIG2494	Pro	Leu
Branch 3	T2626664C	SAPIG2545	Lys	Glu
Branch 3	A2632746T	SAPIG2550	Asn	Lys
Branch 3	C2632949G	SAPIG2550	Gly	Arg
Branch 3	A2660495C	SAPIG2573	Lys	Asn

Branch 3	G2676964A	SAPIG2589	Pro	Ser
Branch 3	C2716556T	SAPIG2625	Val	Ile
Branch 3	G2739946A	SAPIG2651	Met	Ile
Branch 3	G2772435A	SAPIG2678	Ser	Phe
Branch 3	A2773585G	SAPIG2680	Lys	Glu
Branch 3	A2803688T	SAPIG2699	Ile	Asn
Branch 3	G2824157T	SAPIG2712	Thr	Lys
Branch 3	T2831612C	SAPIG2720	Ser	Pro
Branch 3	G2838382T	*hisH*	His	Asn
Branch 3	C2838554A	*hisB*	Gly	Val
Branch 3	C2848009T	*pcp*	Pro	Leu
Branch 3	C2849279A	SAPIG2738	Glu	Asp
Branch 3	G2857671A	SAPIG2743	Gly	Asp
Branch 4	C2864921T	SAPIG2751	Glu	Lys
Branch 4	C158311T	*deoB*	Pro	Ser
Branch 4	G159387A	*phnE1*	Ala	Val
Branch 4	C254208T	SAPIG0231	Thr	Ile
Branch 4	G422734A	SAPIG0406	Gly	Asp
Branch 4	G484168A	SAPIG0468	Ala	Thr
Branch 4	T619496A	SAPIG0593	Gln	Leu
Branch 4	A682708G	*hxlA*	Asp	Gly
Branch 4	T688112A	SAPIG0649	Leu	*
Branch 4	C715450G	SAPIG0681	Tyr	*
Branch 4	A724813G	SAPIG0690	Lys	Glu
Branch 4	T810885G	*pfkB*	Asp	Glu
Branch 4	C864995T	SAPIG0824	Glu	Lys
Branch 4	G969752A	SAPIG0932	Thr	Ile
Branch 4	C992006G	*addB*	Gln	Glu
Branch 4	G1034241A	SAPIG0985	Val	Ile
Branch 4	T1189325A	*pheT*	Leu	Ile
Branch 4	G1193384A	SAPIG1139	Ala	Thr
Branch 4	C1214573T	SAPIG1161	Arg	His
Branch 4	C1371725A	SAPIG1304	His	Asn
Branch 4	G1406509A	SAPIG1342	Ala	Thr
Branch 4	G1447299A	*trpA*	Val	Ile
Branch 4	C1510429T	SAPIG1434	Ala	Thr
Branch 4	C1769675T	SAPIG1687	Thr	Ile
Branch 4	G1812609A	SAPIG1728	His	Tyr
Branch 4	C1816542T	SAPIG1731	Asp	Asn
Branch 4	T1822807C	*thrS*	Ile	Val
Branch 4	T1872964G	*serA*	Ser	Ala
Branch 4	T1874245C	SAPIG1777	Asn	Ser
Branch 4	C1922423T	*leuS*	Glu	Lys
Branch 4	T1936571C	SAPIG1824	Val	Ala
Branch 4	C2067589T	SAPIG1993	Gly	Ser
Branch 4	T2167886G	*ilvD*	Ile	Met

Branch 4	G2257386A	*deoC*	Ala	Val
Branch 4	G2438997T	SAPIG2362	His	Asn
Branch 4	C2516274T	SAPIG2439	Ser	Phe
Branch 4	C2578324T	SAPIG2494	Gly	Asp
Branch 4	A2636096G	SAPIG2553	Ile	Thr
Branch 4	G2659954T	SAPIG2572	Gln	Lys
Branch 4	G2686427T	SAPIG2598	Gly	Cys
Branch 4	A2696538G	SAPIG2608	Asn	Ser
Branch 4	C2773792A	SAPIG2679	Glu	Asp
Branch 4	G2812625T	SAPIG2704	Ser	Tyr
Branch 4	G2817804A	SAPIG2705	Pro	Ser
Branch 4	C2856688T	*rarD*	Gly	Asp
Branch 4	C2862740T	SAPIG2748	Ala	Val

[1]Branch 1 included all the samples in the clade from 2008-60-970 to T4. Branch 2 included eight samples (LA-MSSA-2, LA-MSSA-3, LA-MSSA-10, LA-MSSA-1, LA-MSSA-11, LA-MSSA-8, LA-MSSA-12 and LA-MSSA-4). Branch 3 included four samples (LA-MSSA-14, LA-MSSA-9,LA-MSSA-15 and LA-MSSA-6). Branch 4 included two samples (LA-MSSA-13 and LA-MSSA-7).
[2]The genomic position was mapped to the chromosome of *S. aureus* CC398 reference strain S0385 (GenBank accession no. AM990992).
[3]SNPs noted in Stegger et al. [31] to distinguish ST398 livestock from human isolates.
*Stop codon.

Table S2. Characteristics of isolates used for virulence experiments.

Isolate number/name	Year of isolation	Host	spa type	MLST	MSSA/MRSA type	Primary infection type
CA-MRSA 1	2014	Human-adult	t034	ST398	CA-MRSA	Respiratory
CA-MRSA 2	2014	Human-adult	t034	ST398	CA-MRSA	Respiratory
CA-MRSA 3	2012	Human-adult	t034	ST398	CA-MRSA	Respiratory
CA-MRSA 4	2011	Human-adult	t011	ST398	CA-MRSA	Respiratory
CA-MRSA 5	2012	Human-child	t034	ST398	CA-MRSA	SSTI
CA-MRSA 6	2012	Human-child	t571	ST398	CA-MRSA	SSTI
CA-MRSA 7	2011	Human-adult	t034	ST398	CA-MRSA	SSTI
CA-MSSA 8	2010	Human-adult	t034	ST398	CA-MSSA	Respiratory
CA-MSSA 9	2014	Human-adult	t034	ST398	CA-MSSA	Respiratory
CA-MSSA 10	2011	Human-adult	t571	ST398	CA-MSSA	SSTI
CA-MSSA 11	2014	Human-adult	t571	ST398	CA-MSSA	Respiratory
CA-MSSA 12	2014	Human-adult	t1451	ST398	CA-MSSA	Respiratory
CA-MSSA 13	2014	Human-adult	t3085	ST398	CA-MSSA	SSTI
CA-MSSA 14	2014	Human-adult	t571	ST398	CA-MSSA	Respiratory
CA-MSSA 15	2010	Human-adult	t011	ST398	CA-MSSA	SSTI
CA-MSSA 16	2011	Human-adult	t034	ST398	CA-MSSA	SSTI
CA-MSSA 17	2012	Human-adult	t1451	ST398	CA-MSSA	SSTI
CA-MSSA 18	2014	Human-adult	t034	ST398	CA-MSSA	SSTI
CA-MSSA 19	2010	Human-adult	t1451	ST398	CA-MSSA	Respiratory
CA-MSSA 20	2014	Human-adult	t571	ST398	CA-MSSA	SSTI
CA-MSSA 21	2014	Human-adult	t571	ST398	CA-MSSA	SSTI
CA-MSSA 22	2012	Human-adult	t571	ST398	CA-MSSA	SSTI
CA-MSSA 23	2012	Human-adult	t571	ST398	CA-MSSA	Respiratory
CA-MSSA 24	2012	Human-adult	t1255	ST398	CA-MSSA	SSTI
CA-MSSA 25	2010	Human-adult	t034	ST398	CA-MSSA	SSTI
CA-MSSA 26	2012	Human-child	t034	ST398	CA-MSSA	SSTI
CA-MSSA 27	2012	Human-child	t571	ST398	CA-MSSA	SSTI
CA-MSSA 28	2012	Human-child	t011	ST398	CA-MSSA	Respiratory
CA-MSSA 29	2012	Human-child	t571	ST398	CA-MSSA	SSTI
CA-MSSA 30	2010	Human-adult	t034	ST398	CA-MSSA	Respiratory
CA-MSSA 31	2012	Human-child	t034	ST398	CA-MSSA	Respiratory
CA-MSSA 32	2012	Human-child	t571	ST398	CA-MSSA	Respiratory
CA-MSSA 33	2012	Human-adult	t034	ST398	CA-MSSA	SSTI
CA-MSSA 34	2011	Human-adult	t571	ST398	CA-MSSA	Respiratory
CA-MSSA 35	2014	Human-adult	t571	ST398	CA-MSSA	Respiratory
CA-MSSA 36	2012	Human-adult	t034	ST398	CA-MSSA	Respiratory
CA-MSSA 37	2011	Human-adult	t034	ST398	CA-MSSA	SSTI
CA-MSSA 38	2010	Human-adult	t034	ST398	CA-MSSA	SSTI
CA-MSSA 39	2012	Human-child	t034	ST398	CA-MSSA	SSTI
CA-MSSA 40	2012	Human-child	t011	ST398	CA-MSSA	SSTI

CA-MSSA 41	2012	Human-adult	t034	ST398	CA-MSSA	Respiratory
CA-MSSA 42	2014	Human-adult	t1451	ST398	CA-MSSA	SSTI
CA-MSSA 43	2011	Human-adult	t571	ST398	CA-MSSA	Respiratory
CA-MSSA 44	2011	Human-adult	t571	ST398	CA-MSSA	Respiratory
CA-MSSA 45	2011	Human-adult	t7160	ST398	CA-MSSA	SSTI
CA-MSSA 46	2012	Human-adult	t011	ST398	CA-MSSA	SSTI
CA-MSSA 47	2012	Human-child	t034	ST398	CA-MSSA	SSTI
CA-MSSA 48	2012	Human-child	t571	ST398	CA-MSSA	SSTI
CA-MSSA 49	2014	Human-adult	t1451	ST398	CA-MSSA	SSTI
CA-MSSA 50	2014	Human-adult	t571	ST398	CA-MSSA	SSTI
CA-MSSA 51	2012	Human-adult	t1451	ST398	CA-MSSA	Respiratory
CA-MSSA 52	2014	Human-adult	t034	ST398	CA-MSSA	Respiratory
CA-MSSA 53	2014	Human-adult	t571	ST398	CA-MSSA	Respiratory
CA-MSSA 54	2011	Human-adult	t034	ST398	CA-MSSA	SSTI
CA-MSSA 55	2010	Human-adult	t034	ST398	CA-MSSA	SSTI
CA-MSSA 56	2011	Human-adult	t034	ST398	CA-MSSA	SSTI
CA-MSSA 57	2014	Human-adult	t034	ST398	CA-MSSA	SSTI
CA-MSSA 58	2014	Human-adult	t571	ST398	CA-MSSA	Respiratory
CA-MSSA 59	2012	Human-adult	t034	ST398	CA-MSSA	SSTI
CA-MSSA 60	2011	Human-adult	t571	ST398	CA-MSSA	Respiratory
CA-MSSA 61	2010	Human-adult	t571	ST398	CA-MSSA	SSTI
LA-MSSA-1	2014	Cattle	t011	ST398	LA-MSSA	Bovine mastitis
LA-MSSA-2	2014	Cattle	t3041	ST398	LA-MSSA	Bovine mastitis
LA-MSSA-3	2014	Cattle	t034	ST398	LA-MSSA	Bovine mastitis
LA-MSSA-4	2014	Cattle	t034	ST398	LA-MSSA	Bovine mastitis
LA-MSSA-5	2014	Cattle	t011	ST398	LA-MSSA	Bovine mastitis
LA-MSSA-6	2014	Cattle	t034	ST398	LA-MSSA	Bovine mastitis
LA-MSSA-7	2014	Cattle	t1451	ST398	LA-MSSA	Bovine mastitis
LA-MSSA-8	2014	Cattle	t034	ST398	LA-MSSA	Bovine mastitis
LA-MSSA-9	2014	Cattle	t034	ST398	LA-MSSA	Bovine mastitis
LA-MSSA-10	2015	Cattle	t034	ST398	LA-MSSA	Bovine mastitis
LA-MSSA-11	2015	Cattle	t034	ST398	LA-MSSA	Bovine mastitis
LA-MSSA-12	2015	Cattle	t034	ST398	LA-MSSA	Bovine mastitis
LA-MSSA-13	2015	Cattle	t1451	ST398	LA-MSSA	Bovine mastitis
LA-MSSA-14	2015	Cattle	t034	ST398	LA-MSSA	Bovine mastitis
LA-MSSA-15	2015	Cattle	t034	ST398	LA-MSSA	Bovine mastitis
S0385		Human-adult	t011	ST398	LA-MRSA	Endocarditis
SF8300 (USA300)		Human-adult	t008	ST8	CA-MRSA	SSTI
LAC (USA300)		Human-adult	t008	ST8	CA-MRSA	SSTI
09-140		Human-adult	t037	ST239	HA-MRSA	SSTI
10-69		Human-adult	t030	ST239	HA-MRSA	SSTI
09-521		Human-adult	t037	ST239	HA-MRSA	Respiratory
09-770		Human-adult	t030	ST239	HA-MRSA	Respiratory

05-72	Human-adult	t002	ST5	HA-MRSA	SSTI
09-384	Human-adult	t002	ST5	HA-MRSA	SSTI
10-210	Human-adult	t002	ST5	HA-MRSA	Respiratory
10-292	Human-adult	t002	ST5	HA-MRSA	Respiratory

Table S3. Presence of virulence genes in analyzed isolates.

Isolate number/name	MLST	sak	chp	scn	sa3	*lukSF*	*sea*	*seb*	*sec*	*sed*	*see*	*seg*	*seh*	*sei*	*sej*	*sek*	*sel*	*sem*	*sen*	*seo*	*sep*	*seq*	*mupA*	*sdrC*	*sdrD*	*sdrE*	*arcA*	*qac*	*icaA*	*sasX*	*clfA*	*bsaA*	*eta*	*etb*	*tst*	*hla*	*hlg*	*fnbA*	*sspA*
CA-MRSA 1	ST398	√	√	√	√																			√	√	√			√		√					√	√	√	√
CA-MRSA 2	ST398	√	√	√	√																			√	√	√			√		√					√	√	√	√
CA-MRSA 3	ST398	√	√	√	√																			√	√	√			√		√					√	√	√	√
CA-MRSA 4	ST398	√	√	√	√				√															√	√	√			√		√					√	√	√	√
CA-MRSA 5	ST398	√	√	√	√																			√	√	√			√		√					√	√	√	√
CA-MRSA 6	ST398	√		√	√																			√	√				√		√					√	√	√	√
CA-MRSA 7	ST398	√	√	√	√																			√	√	√			√		√					√	√	√	√
CA-MSSA 8	ST398					√																		√	√	√		√	√		√					√	√	√	√
CA-MSSA 9	ST398	√		√	√																√			√	√	√			√		√					√	√	√	√
CA-MSSA 10	ST398	√		√	√		√																	√	√	√			√		√					√	√	√	√
CA-MSSA 11	ST398	√		√	√																					√			√		√					√	√	√	√
CA-MSSA 12	ST398	√		√	√	√																		√	√	√			√		√					√	√	√	√
CA-MSSA 13	ST398	√		√	√			√									√							√	√	√			√		√					√	√	√	√
CA-MSSA 14	ST398	√		√	√				√															√	√				√		√					√	√	√	√
CA-MSSA 15	ST398	√	√	√	√																			√	√	√			√		√					√	√	√	√
CA-MSSA 16	ST398	√	√	√	√																			√		√			√		√					√	√	√	√
CA-MSSA 17	ST398	√	√	√	√	√																		√	√				√		√					√	√	√	√
CA-MSSA 18	ST398	√	√	√	√																				√	√			√		√					√	√	√	√
CA-MSSA 19	ST398	√		√	√																			√	√	√			√		√					√	√	√	√
CA-MSSA 20	ST398	√		√	√																			√	√	√			√		√					√	√	√	√
CA-MSSA 21	ST398	√		√	√																			√	√	√			√		√					√	√	√	√
CA-MSSA 22	ST398	√		√	√				√															√	√				√		√					√	√	√	√
CA-MSSA 23	ST398	√		√	√																			√	√	√			√		√					√	√	√	√
CA-MSSA 24	ST398	√	√	√	√																			√	√	√			√		√					√	√	√	√

CA-MSSA 25	ST398			√	√									√										√	√	√			√		√					√	√	√	√
CA-MSSA 26	ST398			√												√								√	√	√			√		√					√	√	√	√
CA-MSSA 27	ST398	√		√	√																√			√	√	√			√		√					√	√	√	√
CA-MSSA 28	ST398			√	√																			√	√	√			√		√					√	√	√	√
CA-MSSA 29	ST398	√		√	√																				√	√			√		√					√	√	√	√
CA-MSSA 30	ST398	√	√	√	√	√																		√	√				√		√					√	√	√	√
CA-MSSA 31	ST398	√	√	√	√			√				√	√				√							√	√	√			√		√				√	√	√	√	√
CA-MSSA 32	ST398	√		√	√																			√	√				√		√					√	√	√	√
CA-MSSA 33	ST398	√	√	√	√				√															√	√	√			√		√					√	√	√	√
CA-MSSA 34	ST398	√		√	√																			√	√	√			√		√					√	√	√	√
CA-MSSA 35	ST398	√		√	√												√							√	√	√			√		√					√	√	√	√
CA-MSSA 36	ST398	√	√	√	√																				√	√			√		√					√	√	√	√
CA-MSSA 37	ST398	√	√	√	√																			√	√	√			√		√					√	√	√	√
CA-MSSA 38	ST398	√	√	√	√	√																		√	√	√			√		√					√	√	√	√
CA-MSSA 39	ST398																							√	√	√			√		√					√	√	√	√
CA-MSSA 40	ST398	√		√	√																			√	√	√			√		√					√	√	√	√
CA-MSSA 41	ST398	√	√	√	√																			√		√			√		√					√	√	√	√
CA-MSSA 42	ST398	√		√	√																			√	√	√			√		√					√	√	√	√
CA-MSSA 43	ST398	√	√	√	√				√															√	√				√		√					√	√	√	√
CA-MSSA 44	ST398	√		√	√	√																		√	√	√			√		√					√	√	√	√
CA-MSSA 45	ST398	√		√	√																			√	√	√			√		√					√	√	√	√
CA-MSSA 46	ST398	√	√	√	√																								√		√					√	√	√	√
CA-MSSA 47	ST398																							√	√	√			√		√					√	√	√	√
CA-MSSA 48	ST398	√		√	√																			√	√	√		√	√		√					√	√	√	√
CA-MSSA 49	ST398	√		√	√		√	√																√	√				√		√					√	√	√	√
CA-MSSA 50	ST398	√		√	√	√			√												√			√	√	√			√		√					√	√	√	√
CA-MSSA 51	ST398	√		√	√									√										√	√	√			√		√					√	√	√	√
CA-MSSA 52	ST398	√		√	√		√																	√	√				√		√					√	√	√	√

CA-MSSA 53	ST398	√		√	√																			√	√			√		√					√	√	√	√
CA-MSSA 54	ST398	√		√	√	√																		√	√	√			√		√					√	√	√	√
CA-MSSA 55	ST398	√	√	√	√									√		√						√	√	√	√		√	√		√	√			√	√	√	√	√
CA-MSSA 56	ST398	√	√	√	√																			√		√			√		√					√	√	√	√
CA-MSSA 57	ST398	√	√	√	√	√												√								√				√		√					√	√	√	√
CA-MSSA 58	ST398	√	√	√	√		√		√															√	√	√			√		√					√	√	√	√
CA-MSSA 59	ST398	√	√	√	√																			√	√	√			√		√					√	√	√	√
CA-MSSA 60	ST398	√		√	√																			√	√	√			√		√					√	√	√	√
CA-MSSA 61	ST398	√	√	√	√												√								√	√			√		√					√	√	√	√
LA-MSSA-1	ST398	√	√	√	√																			√	√	√			√		√					√	√	√	√
LA-MSSA-2	ST398																						√		√			√		√					√	√	√	√
LA-MSSA-3	ST398																						√	√				√		√					√	√	√	√
LA-MSSA-4	ST398																						√	√	√			√		√					√	√	√	√
LA-MSSA-5	ST398																							√				√		√					√	√	√	√
LA-MSSA-6	ST398																						√	√	√			√		√					√	√	√	√
LA-MSSA-7	ST398		√	√	√																				√				√		√					√	√	√	√
LA-MSSA-8	ST398																						√	√	√			√		√					√	√	√	√
LA-MSSA-9	ST398																						√	√	√			√		√					√	√	√	√
LA-MSSA-10	ST398																						√		√			√		√					√	√	√	√
LA-MSSA-11	ST398																						√	√	√			√		√					√	√	√	√
LA-MSSA-12	ST398																						√	√				√		√					√	√	√	√
LA-MSSA-13	ST398		√	√	√																			√	√	√			√		√					√	√	√	√
LA-MSSA-14	ST398																							√	√			√		√					√	√	√	√
LA-MSSA-15	ST398																						√		√			√		√					√	√	√	√
S0385	ST398																						√	√				√		√					√	√	√	√
SF8300 (USA300)	ST8	√	√	√	√	√											√						√		√	√	√	√		√		√	√				√	√	√	√
LAC (USA300)	ST8	√	√	√	√	√											√						√		√	√	√	√		√		√	√				√	√	√	√
09-140	ST239	√	√	√	√		√											√						√		√	√	√			√	√	√	√				√	√	√	√

10-69	ST239	√	√	√	√		√									√						√	√	√	√	√		√	√		√	√				√	√	√	√
09-521	ST239	√	√	√	√		√									√						√		√	√	√			√	√	√	√				√	√	√	√
09-770	ST239	√	√	√	√		√									√						√		√	√	√			√	√	√	√				√	√	√	√
05-72	ST5	√	√	√	√							√		√			√	√	√	√					√				√	√	√				√	√	√	√	√
09-384	ST5	√	√	√	√							√		√			√	√	√	√					√				√		√				√	√	√	√	√
10-210	ST5	√	√	√	√							√		√			√	√	√	√					√				√	√	√				√	√	√	√	√
10-292	ST5	√	√	√	√							√		√			√	√	√	√			√			√			√		√				√	√	√	√	√

Table S4. Presence of antibiotic resistance in analyzed isolates.

Isolate number/name	MLST	MSSA/MRSA type	SCCmec	Gentamicin	Penicillin	Cefoxitin	Cefazolin	Levofloxacin	Erythromycin	Clindamycin	TMP/SMX	Fosfomycin	Rifampicin	Teicoplanin	Vancomycin	Linezolid
CA-MRSA 1	ST398	CA-MRSA	V		√	√	√		√							
CA-MRSA 2	ST398	CA-MRSA	V		√	√			√							
CA-MRSA 3	ST398	CA-MRSA	V		√	√	√		√							
CA-MRSA 4	ST398	CA-MRSA	V		√	√	√		√							
CA-MRSA 5	ST398	CA-MRSA	IV		√	√	√									
CA-MRSA 6	ST398	CA-MRSA	V		√	√	√		√	√						
CA-MRSA 7	ST398	CA-MRSA			√	√	√		√							
CA-MSSA 8	ST398	CA-MSSA			√											
CA-MSSA 9	ST398	CA-MSSA			√				√							
CA-MSSA 10	ST398	CA-MSSA							√		√					
CA-MSSA 11	ST398	CA-MSSA		√	√				√	√						
CA-MSSA 12	ST398	CA-MSSA			√					√						
CA-MSSA 13	ST398	CA-MSSA			√											
CA-MSSA 14	ST398	CA-MSSA			√				√							
CA-MSSA 15	ST398	CA-MSSA			√				√							
CA-MSSA 16	ST398	CA-MSSA			√			√	√	√						
CA-MSSA 17	ST398	CA-MSSA		√	√				√	√						
CA-MSSA 18	ST398	CA-MSSA			√			√	√	√						
CA-MSSA 19	ST398	CA-MSSA			√				√							
CA-MSSA 20	ST398	CA-MSSA			√											
CA-MSSA 21	ST398	CA-MSSA			√				√	√						
CA-MSSA 22	ST398	CA-MSSA														

CA-MSSA 23	ST398	CA-MSSA	√	√				√			
CA-MSSA 24	ST398	CA-MSSA		√				√			
CA-MSSA 25	ST398	CA-MSSA		√				√		√	
CA-MSSA 26	ST398	CA-MSSA		√				√			
CA-MSSA 27	ST398	CA-MSSA		√				√			
CA-MSSA 28	ST398	CA-MSSA		√				√			
CA-MSSA 29	ST398	CA-MSSA	√	√				√	√		
CA-MSSA 30	ST398	CA-MSSA		√					√		
CA-MSSA 31	ST398	CA-MSSA		√			√	√	√		
CA-MSSA 32	ST398	CA-MSSA	√	√				√			
CA-MSSA 33	ST398	CA-MSSA		√			√	√			
CA-MSSA 34	ST398	CA-MSSA		√			√	√	√		
CA-MSSA 35	ST398	CA-MSSA	√	√					√		
CA-MSSA 36	ST398	CA-MSSA		√			√	√			
CA-MSSA 37	ST398	CA-MSSA	√	√				√	√		
CA-MSSA 38	ST398	CA-MSSA		√			√	√	√		
CA-MSSA 39	ST398	CA-MSSA									
CA-MSSA 40	ST398	CA-MSSA						√			
CA-MSSA 41	ST398	CA-MSSA		√							
CA-MSSA 42	ST398	CA-MSSA	√								
CA-MSSA 43	ST398	CA-MSSA	√	√				√			
CA-MSSA 44	ST398	CA-MSSA		√				√	√		
CA-MSSA 45	ST398	CA-MSSA		√			√	√	√		√
CA-MSSA 46	ST398	CA-MSSA	√	√				√			
CA-MSSA 47	ST398	CA-MSSA		√				√	√		
CA-MSSA 48	ST398	CA-MSSA		√			√			√	
CA-MSSA 49	ST398	CA-MSSA		√				√	√		
CA-MSSA 50	ST398	CA-MSSA		√			√				√

CA-MSSA 51	ST398	CA-MSSA		√	√							√	
CA-MSSA 52	ST398	CA-MSSA		√	√				√	√			
CA-MSSA 53	ST398	CA-MSSA							√		√		
CA-MSSA 54	ST398	CA-MSSA							√				
CA-MSSA 55	ST398	CA-MSSA			√				√	√			
CA-MSSA 56	ST398	CA-MSSA			√				√			√	√
CA-MSSA 57	ST398	CA-MSSA			√			√	√	√			
CA-MSSA 58	ST398	CA-MSSA		√					√		√	√	
CA-MSSA 59	ST398	CA-MSSA			√			√	√	√			
CA-MSSA 60	ST398	CA-MSSA		√	√							√	
CA-MSSA 61	ST398	CA-MSSA			√					√			
LA-MSSA-1	ST398	LA-MSSA			√				√				
LA-MSSA-2	ST398	LA-MSSA			√				√				
LA-MSSA-3	ST398	LA-MSSA			√				√	√			
LA-MSSA-4	ST398	LA-MSSA			√				√	√			
LA-MSSA-5	ST398	LA-MSSA			√								
LA-MSSA-6	ST398	LA-MSSA		√	√				√				
LA-MSSA-7	ST398	LA-MSSA							√				
LA-MSSA-8	ST398	LA-MSSA		√	√				√	√			
LA-MSSA-9	ST398	LA-MSSA		√	√				√	√			
LA-MSSA-10	ST398	LA-MSSA			√				√	√			
LA-MSSA-11	ST398	LA-MSSA		√	√				√	√			
LA-MSSA-12	ST398	LA-MSSA			√				√	√			
LA-MSSA-13	ST398	LA-MSSA							√				
LA-MSSA-14	ST398	LA-MSSA		√	√				√				
LA-MSSA-15	ST398	LA-MSSA		√	√				√	√			
S0385	ST398	LA-MRSA	V		√	√	√	√					
SF8300 (USA300)	ST8	CA-MRSA	IV		√	√	√		√				

LAC (USA300)	ST8	CA-MRSA			√	√	√	√	√				
09-140	ST239	HA-MRSA	III	√	√	√	√	√	√	√		√	√
10-69	ST239	HA-MRSA	III	√	√	√	√	√	√	√		√	√
09-521	ST239	HA-MRSA	III	√	√	√	√	√	√	√			√
09-770	ST239	HA-MRSA	III	√	√	√	√	√	√	√			√
05-72	ST5	HA-MRSA	II	√	√	√	√	√	√			√	
09-384	ST5	HA-MRSA	II	√	√	√	√	√	√	√		√	√
10-210	ST5	HA-MRSA	II	√	√	√	√	√	√	√		√	
10-292	ST5	HA-MRSA	II	√	√	√	√	√	√	√		√	

Table S5. Non-synonymous SNPs (as compared to the MRSA ancestor node and the closest MSSA neighbors) in ST398 CA-MRSA isolates

Isolates with mutations present	Number of genes	Gene ID	Gene name	Function	aa[1] change (from – to) in isolates							
					1-4		5		6		7	
1/2/3/4/7	5	SAPIG0728	*dhaK*	dihydroxyacetone kinase, DhaK subunit	Leu	Phe					Asp	Asn
		SAPIG0769		FAD binding domain of DNA photolyase protein	Thr	Ala					Gln	Arg
		SAPIG1757		DHH family protein	Glu	Lys					Glu	Ala
		SAPIG2096	*leuA*	2-isopropylmalate synthase	His	Tyr					Thr	Ile
		SAPIG2209	*glmS*	glutamine-fructose-6-phosphate transaminase (isomerizing)	Ile	Val					Asp	Asn
5/6	3	SAPIG0925		cytosol aminopeptidase family protein			Gly	Ser	Thr	Ala		
		SAPIG1434		EbhA protein			His	Arg	Leu	Pro		
		SAPIG1996	*putP*	sodium/proline symporter			Gly	Asp	Leu	Phe		
5/7	5	SAPIG2732		polysaccharide deacetylase domain protein			His	Gln			Val	Asp
		SAPIG2654		fructose-bisphosphate aldolase class-I			Gly	Ser			Asn	Ser
		SAPIG1581		(Dihydrolipoyllysine-residue (2-methylpropanoyl)transferase) (E2)			Thr	Asn			Ser	Thr
		SAPIG1428	*thyA*	thymidylate synthase			Phe	Tyr			Ala	Thr
		SAPIG2381		amidohydrolase subfamily			Arg	Lys			Glu	Lys
6/7	3	SAPIG0912		HAD-superfamily subfamily IIA hydrolase					Gly	Glu	Ser	Pro
		SAPIG1584	*IpdA*	dihydrolipoyl dehydrogenase					Lys	Glu	Pro	Ser
		SAPIG1256	*hsIU*	heat shock protein HslVU, ATPase subunit HslU					Arg	Lys	Thr	Ala
1/2/3/4	24	SAPIG0244		acyl-CoA synthetase	Ala	Thr						
		SAPIG0702		Na(+)/H(+) antiporter subunit A (Mrp complex subunit A)	Ala	Thr						
		SAPIG0746		HTH-type transcriptional regulator SarX (Staphylococcal accessory regulator X)	Ser	Thr						
		SAPIG0999		adenylate cyclase	Pro	Ser						
		SAPIG1054		beta-lactamase	Lys	Asn						
		SAPIG1118		conserved hypothetical protein	Glu	Lys						
		SAPIG1186		protein YlmE	Asp	Asn						
		SAPIG1242		membrane protein, putative	Val	Leu						
		SAPIG1245		succinyl-CoA synthetase beta chain (SCS-alpha) (VEG63)	Asn	Ser						
		SAPIG1446		protein YpsA	Glu	Stop						
		SAPIG1592	*accC*	acetyl-CoA carboxylase, biotin carboxylase	Asp	Tyr						
		SAPIG1670		biotin carboxylase (Acetyl-CoA carboxylase subunit A) (ACC)	Thr	Ala						
		SAPIG1800		metallo-beta-lactamase superfamily protein	Asp	Gly						
		SAPIG1925		regulatory protein	Asn	Ser						
		SAPIG1980		conserved hypothetical	Ala	Val						

				protein				
		SAPIG2069		serine-aspartate repeat family protein, SdrH	Pro	Leu		
		SAPIG2262		hyaluronate lyase (Hyaluronidase) (HYase)	Asn	Asp		
		SAPIG2372		sodium/bile acid symporter family protein	Ala	Val		
		SAPIG2450		nitrate reductase, alpha subunit	Ile	Ser		
		SAPIG2461		lipoprotein, putative	Gly	Val		
		SAPIG2631		hydrolase, alpha/beta hydrolase fold family	Tyr	His		
		SAPIG2644		hydrolase, CocE/NonD family	Gly	Ser		
		SAPIG2708		conserved hypothetical protein	Asn	Asp		
		SAPIG2733		YceI like family	Ser	Asn		
5	41	SAPIG0199	*agrC*	N-acetyl-gamma-glutamyl-phosphate reductase			Thr	Ser
		SAPIG0461		hypothetical symporter YdjN			Arg	Cys
		SAPIG0523		conserved hypothetical protein			Asp	Tyr
		SAPIG0636		serine-aspartate repeat-containing protein C			Glu	Gly
		SAPIG0665	*mvaD*	diphosphomevalonate decarboxylase			Ala	Thr
		SAPIG0705		MrpD			Asp	Asn
		SAPIG0786		membrane protein, putative			Gly	Arg
		SAPIG0808		ribonucleoside-diphosphate reductase, alpha subunit			His	Tyr
		SAPIG0847		NAD-dependent malic enzyme (NAD-ME)			Leu	Phe
		SAPIG0848		conserved hypothetical protein			Leu	Phe
		SAPIG0854	*gpml*	2,3-bisphosphoglycerate-independent phosphoglycerate mutase			Val	Met
		SAPIG1123		conserved hypothetical protein			Asp	Tyr
		SAPIG1128		iron-regulated protein			Glu	Val
		SAPIG1219		protein kinase			Glu	Lys
		SAPIG1225		DAK2 domain protein			Ile	Val
		SAPIG1252	*topA*	DNA topoisomerase I			Gly	His
		SAPIG1348		exonuclease SbcC			Asn	Asp
		SAPIG1385		transposase			Asp	Asn
		SAPIG1415	*sucA*	oxoglutarate dehydrogenase (succinyl-transferring), E1 component			Ile	Met
		SAPIG1421		carboxy-processing protease			Gly	Ser
		SAPIG1573		oligo-1,6-glucosidase (Oligosaccharide alpha-1,6-glucosidase)			Arg	His
		SAPIG1623		ATP-dependent RNA helicase			Ala	Val
		SAPIG1681		protein Stu1959			Val	Ile
		SAPIG1684		TPR domain protein			Ala	Val
		SAPIG1692		HesA/MoeB/ThiF family protein			Met	Ile
		SAPIG1728		probable GTP-binding protein EngB			Ile	Val
		SAPIG1783		iron-regulated surface determinant protein H (SA surface protein I)			Pro	Ser

		SAPIG1792		conserved protein YtxG	Val	Ala		
		SAPIG1868		conserved hypothetical protein	Asp	Asn		
		SAPIG2062		probable succinyl-diaminopimelate desuccinylase (sdap)	Gly	Arg		
		SAPIG2210		pts system mannitol-specific eiicba component (eiicba-mtl) (eii-mtl)	Lys	[illegible]		
		SAPIG2261		MerR family regulatory protein	Trp	Stop		
		SAPIG2326		molybdenum cofactor biosynthesis protein B	Val	Leu		
		SAPIG2377		Na+/H+ antiporter family protein	Val	Leu		
		SAPIG2422		TpgX protein	Ala	Ser		
		SAPIG2454		transcriptional regulator NirR	Arg	Leu		
		SAPIG2470		immunoglobulin G-binding protein	Gln	Stop		
		SAPIG2514		dipeptide transport ATP-binding protein DppD	Ala	Thr		
		SAPIG2523		putative efflux pump component MtrF	Ile	Leu		
		SAPIG2585		esterase, putative	Gly	Arg		
		SAPIG2643		hydrolase, CocE/NonD family	Arg	Lys		
6	32	SAPIG0136		conserved hypothetical protein			Asp	Asn
		SAPIG0213		type I site-specific deoxyribonuclease, HsdR family subfamily, putative			Ala	Thr
		SAPIG0238		glycerophosphoryl diester phosphodiesterase			Gln	Stop
		SAPIG0256		transcriptional antiterminator, BglG family			Gln	Pro
		SAPIG0279		PTS system, IIA component			Phe	Ser
		SAPIG0460		NADPH-dependent oxidoreductase			Phe	Leu
		SAPIG0547		Orn/Lys/Arg decarboxylase			Pro	Leu
		SAPIG0613	*rplA*	ribosomal protein L1			Gly	Ala
		SAPIG0642		conserved protein, Carbohydrate Esterase Family 14			Gly	Arg
		SAPIG0668		probable pyridine nucleotide-disulfide oxidoreductase YkgC			Val	Met
		SAPIG0814	*murB*	UDP-N-acetylenolpyruvoylglucosamine reductase			Pro	Leu
		SAPIG0844		conserved hypothetical protein			Ile	Thr
		SAPIG0856		conserved hypothetical protein			Asp	Gly
		SAPIG0904		2-nitropropane dioxygenase			Gly	Arg
		SAPIG0989		oligopeptide transport ATP-binding protein OppD			Gly	Glu
		SAPIG1214	*def*	polypeptide deformylase			Met	Ile
		SAPIG1361	*CAQ*	FmtC			Ala	Val
		SAPIG1365	*CAQ*	ImpB/MucB/SamB family protein			Ser	Arg
		SAPIG1464	*aroA*	3-phosphoshikimate 1-carboxyvinyltransferase			Gly	Ser

		SAPIG1703	*queA*	S-adenosylmethionine:tRNA ribosyltransferase-isomerase	Val	Ile		
		SAPIG1731		conserved hypothetical protein	Glu	Lys		
		SAPIG1808		conserved hypothetical protein	Val	Ala		
		SAPIG1836		2,5-diketo-D-gluconic acid reductase A	Asp	Glu		
		SAPIG1998	*ligA*	DNA ligase, NAD-dependent	Lys	Thr		
		SAPIG2087		DNA mismatch repair protein MutS	Ala	Thr		
		SAPIG2142	*murA*	UDP-N-acetylglucosamine 1-carboxyvinyltransferase	Thr	Ile		
		SAPIG2215		truncated FmtB protein	Thr	Lys		
		SAPIG2344	*ureD*	urease accessory protein UreD	Val	Ala		
		SAPIG2484		glycerate kinase	Asp	Asn		
		SAPIG2499		OpuCB	Ala	Thr		
		SAPIG2511		probable membrane protein	Gln	Lys		
		SAPIG2521		conserved hypothetical protein	Ala	Thr		
7	98	SAPIG0151		probable membrane transport protein			His	Gln
		SAPIG0184		conserved hypothetical protein			Glu	Lys
		SAPIG0193		surfactin/siderophore synthetase			Arg	Cys
		SAPIG0224		maltose ABC transporter substrate-binding protein			His	Tyr
		SAPIG0240		staphylocoagulase			Asn	Lys
		SAPIG0242		3-hydroxyacyl-CoA dehydrogenase			Asp	Ala
		SAPIG0247		membrane protein, putative			Glu	Val
		SAPIG0258		PTS system galactitol-specific enzyme II B component			Pro	Leu
		SAPIG0262		hexitol dehydrogenase			His	Asp
		SAPIG0267		cdp-glycerol:poly(glycerophosphate) glycerophosphotransFerase			Met	Val
		SAPIG0285		ribose transporter RbsU			Pro	His
		SAPIG0304		conserved hypothetical protein			Gln	Stop
		SAPIG0305		conserved hypothetical protein			Gly	Arg
		SAPIG0322		lipoprotein-releasing system ATP-binding protein LolD			Leu	Phe
		SAPIG0421		membrane protein			Lys	Gln
		SAPIG0466		xanthine permease			Gly	Ala
		SAPIG0502		gram-positive signal peptide, ysirk family protein			Glu	Lys
		SAPIG0507		staphylococcus tandem lipoprotein			Thr	Ala
		SAPIG0521		conserved hypothetical protein			Ile	Thr
		SAPIG0563		endoribonuclease L-PSP, putative			Glu	Val
		SAPIG0569	*mfd*	transcription-repair coupling factor			Ile	Val
		SAPIG0593		transcriptional regulator of GntR family			Glu	Lys
		SAPIG0624		thermostable carboxypeptidase 1			Val	Gly

SAPIG0626		chaperone protein HchA (Hsp31) (EcHsp31)	Thr	Met
SAPIG0637		Ser-Asp rich fibrinogen-binding, bone sialoprotein-binding protein	Thr	Ala
SAPIG0658		amino acid permease	Gly	Arg
SAPIG0675		conserved hypothetical protein	Ala	Val
SAPIG0678		staphylococcus aureus paralogous family	Thr	Thr
SAPIG0683		acetyltransferase, gnat family	Asp	Tyr
SAPIG0686		alcohol dehydrogenase, propanol-preferring	Val	Phe
SAPIG0739		bacitracin export ATP-binding protein BceA	Ser	Ile
SAPIG0745		transcriptional regulator, AraC family	Asp	Glu
SAPIG0747		conserved hypothetical protein	Gly	Arg
SAPIG0756		conserved hypothetical protein	Asp	Tyr
SAPIG0781		bactoprenol glucosyl transferase	Gly	Ala
SAPIG0828		ribosomal subunit interface protein	Ala	Val
SAPIG0864		acetyltransferase, gnat family	Glu	Lys
SAPIG0893		ABC transporter, permease protein	Leu	Ile
SAPIG0920		conserved protein YuzD	Ala	Glu
SAPIG0928		conserved hypothetical protein	Gly	Gly
SAPIG0929		MrpG	Val	Ile
SAPIG0985		oligopeptide transport ATP-binding protein OppF	Arg	Cys
SAPIG0997		protein (Fragment)	Ala	Thr
SAPIG1001		GTP pyrophosphokinase	Thr	Ser
SAPIG1047		putative aminotransferase A	Asp	Val
SAPIG1053		cell envelope-related transcriptional attenuator domain family	Val	Ile
SAPIG1110		cell cycle protein	Met	Ile
SAPIG1212		conserved hypothetical protein	Asn	Asn
SAPIG1217		radical SAM enzyme, Cfr family	Ala	Ser
SAPIG1231		hypothetical protein	Leu	Met
SAPIG1247		LytN protein	Val	Leu
SAPIG1298	*mutS*	DNA mismatch repair protein MutS	Asp	Asn
SAPIG1335		lysine-specific permease	Lys	Glu
SAPIG1363		regulatory protein MsrR	Gln	Pro
SAPIG1368		anthranilate synthase component I	Gly	Ser
SAPIG1394		aspartokinase 2 (Aspartokinase II) (Aspartate kinase 2)	Ala	Thr
SAPIG1426		DegVA	Gly	Ser
SAPIG1430		conserved virulence factor C	Thr	Arg
SAPIG1453		DNA replication initiation protein	Met	Ile
SAPIG1467		nucleoside diphosphate kinase (NDK) (NDP kinase)(Nucleoside-2-P kinase)	Pro	Thr
SAPIG1555		conserved hypothetical	Leu	Ile

		protein		
SAPIG1576	*gnd*	6-phosphogluconate dehydrogenase, decarboxylating	Asp	Gly
SAPIG1585	*recN*	DNA repair protein RecN	Arg	Ser
SAPIG1614		rhomboid family protein	Gln	Arg
SAPIG1622		probable endonuclease 4 (Endonuclease IV)(Endodeoxyribonuclease IV)	Pro	Ser
SAPIG1738		primosomal protein DnaI	Pro	Thr
SAPIG1744		DNA polymerase I (POL I)	Gly	Asp
SAPIG1758		CBS domain protein	Arg	Ile
SAPIG1766		thiol peroxidase Tpx	Met	Leu
SAPIG1771		GAF domain protein	Ala	Val
SAPIG1809		lpxtg-motif cell wall anchor domain	Lys	Glu
SAPIG1824		arsenical pump membrane protein (Arsenic efflux pump protein)	Ala	Val
SAPIG1909		conserved hypothetical protein	Asp	Tyr
SAPIG1917	*fumC*	fumarate hydratase, class II	Ala	Val
SAPIG1982		protein in map 5'region	Ser	Pro
SAPIG2007	*nadE*	NAD+ synthetase	Gly	Ser
SAPIG2058	*map*	map protein, programmed	Ala	Thr
SAPIG2066	*groL*	chaperonin GroL	Pro	Ser
SAPIG2103		zinc metalloprotease	Lys	Stop
SAPIG2106	*rsbW*	anti-sigma B factor	Gly	Ser
SAPIG2123		UDP-N-acetylmuramoyl-tripeptide--D-alanyl-D-alanine ligase	Asn	Ile
SAPIG2158		Sua5/YciO/YrdC/YwlC family protein	Pro	Arg
SAPIG2173		conserved hypothetical protein	Lys	Glu
SAPIG2179	*deoC*	deoxyribose-phosphate aldolase	Met	Ile
SAPIG2230		hemolysin III	Asn	Lys
SAPIG2357		bifunctional autolysin	Asp	Gly
SAPIG2371		phosphoglycolate phosphatase	Gly	Ser
SAPIG2393		ABC-type Na+ efflux pump permease component	Ala	Pro
SAPIG2394		ABC transporter ATP-binding protein	Arg	Cys
SAPIG2406		TetR family regulatory protein	Ile	Phe
SAPIG2412		ABC transporter, ATP-binding protein	Gln	Stop
SAPIG2439		HTH-type transcriptional regulator SarZ (SA accessory regulator Z)	Lys	Stop
SAPIG2548		probable membrane protein	Cys	Tyr
SAPIG2613		dehydrosqualene synthase (Diapophytoene synthase) (DAP synthase)	Arg	Trp
SAPIG2649	*budA*	alpha-acetolactate decarboxylase	Gln	Arg
SAPIG2668		sulfite reductase [NADPH] flavoprotein, alpha-component	Ile	Thr
SAPIG2707		conserved hypothetical protein	Asp	His

	SAPIG2740	collagen adhesin	Asp	Gly

[1]aa, amino acid

Table S6. Indel genetic changes in protein-coding regions of ST398 CA-MRSA isolates

Isolate	Mutation	Significance	Annotation	Gene product
1/2/3/4	907624-907635del	4 aa[1] absent	rnr|indel	ribonuclease R
5	91039insG	frameshift	SAPIG0087|indel	trans-Golgi membrane protein p230
	2336091delT	frameshift	SAPIG2248|indel	cell surface hydrolase
	2575370insC	frameshift	SAPIG2492|indel	amino acid permease
6	670186-670197del	4 aa absent	SAPIG0636|indel	Sdr family protein
	1936800delC	frameshift	SAPIG1824|indel	Arsenic efflux pump protein
	2317268delA		SAPIG2232|indel	conserved hypothetical protein
7	149906-149917del	4 aa absent	SAPIG0145|indel	O-antigen polymerase
	914583-914596del	frameshift	SAPIG0866|indel SAPIG0865|indel	clumping factor A
	1039694insA	frameshift	SAPIG0990|indel	oligopeptide transport ATP-binding protein OppF
	1372852delT	frameshift	hfq|indel	RNA chaperone Hfq
	1507305delT	frameshift	SAPIG1434|indel	EbhA protein
	2265355-2265357del	1 aa absent	SAPIG2188|indel	zinc transporter ZitB

[1]aa, amino acid

Table S7. ORFs in the novel ST398 SCC*mec* type

Location	Strand	Protein size (aa[1])	Gene	Synonym	Product
33290..33769	+	159	*orfX*	orf00001	open reading frame X
34015..34320	+	101	-	orf00002	hypothetical protein
34518..35381	+	287	-	orf00003	hypothetical protein
35489..36964	+	491	-	orf00004	hypothetical protein
37191..38291	+	366	-	orf00005	hypothetical protein
38284..38655	+	123	-	orf00006	hypothetical protein
38652..40295	+	547	-	orf00007	primase putative primase
40249..40365	+	38	-	orf00008	hypothetical protein
40523..42199	+	558	*ccrC1*	orf00009	cassette chromosome recombinase C7
42302..42643	+	113	-	orf00010	hypothetical protein
42739..43050	+	103	-	orf00011	hypothetical protein
43066..43572	+	168	-	orf00012	hypothetical protein
43721..44395	-	224	*tnp*	orf00013	integrase transposase for insertion sequence-like element IS431mec
44653..44820	+	55	-	orf00014	HMG-CoA synthase
45657..46400	+	247	-	orf00015	hypothetical protein V048_02620
46497..46925	+	142	-	orf00016	hypothetical protein
46971..48977	-	668	*mecA*	orf00017	penicillin-binding protein 2'
49000..49110	+	36	*mecR1'*	orf00018	truncated methicillin resistant regulator
49225..49368	+	47	-	orf00019	transposase transposase for IS431mec
49648..49899	+	83	-	orf00020	transposase for insertion sequence-like element IS431mec
50092..50110	+	19	-	DR3	

[1]aa, amino acids
